# Perceptions and use of traditional African medicine in Lubumbashi, Haut-Katanga province (DR Congo): A cross-sectional study

**DOI:** 10.1371/journal.pone.0276325

**Published:** 2022-10-18

**Authors:** Cedrick S. Mutombo, Salvius A. Bakari, Vianney N. Ntabaza, Amandine Nachtergael, Jean-Baptiste S. Lumbu, Pierre Duez, Joh B. Kahumba

**Affiliations:** 1 Laboratory of Pharmacognosy, Department of Pharmacology, Faculty of Pharmaceutical Sciences, Université de Lubumbashi, Lubumbashi, DR Congo; 2 Unit of Therapeutic Chemistry and Pharmacognosy, Faculty of Medicine and Pharmacy, University of Mons, Mons, Belgium; 3 Service de Chimie Organique, Département de Chimie, Faculté des Sciences, Université de Lubumbashi, Lubumbashi, DR Congo; Flinders University, AUSTRALIA

## Abstract

In the Democratic Republic of Congo, the desire of the Ministry of Health to integrate Traditional African Medicine into the Official Health System remains limited by the lack of reliable data on several aspects of this medicine. This study aims to determine the perceptions of the Lubumbashi population towards Traditional African Medicine and the contexts of recourse to these therapeutic modalities. We conducted semi-structured interviews of population samples in each of the 7 Lubumbashi municipalities, which were semi-randomly selected in proportions to each population size, from January to June 2017 and from February to July 2018. A total of 4278 people (average age, 32.1 ± 10.4 years; 36.5% of women) have been surveyed. Among them, 75.8% define "Traditional African Medicine" as "herbal-based treatments"; 79.4% have resorted to traditional medicine, for several pathologies and social problems. This medicine was preferred for efficiency, speed of healing and low cost, as well as the presence of the diseases against which it would be the only one used. Most, (52.1%) have started with Conventional Medicine, then resorted to Traditional African Medicine, 34.7% started directly with Traditional African Medicine, while 13.2% combined these two medicines. From those who have resorted to Traditional African Medicine (n = 3396), 55% declare no concern towards traditional medicine, while 42.5% fear looseness, overdose, intoxication, and lack of hygiene; from those who have not resorted to Traditional African Medicine (n = 882), 78% are fearful of fear looseness, witchcraft, and fetishism. Traditional African Medicine remains an important health care resource for the Lubumbashi people. It is essential that decision-makers consider the importance of this health sector for the population, but also the reported fears and hindrances, and work towards the regulation, sanitization, and control of this medicine to ensure its safe use.

## Introduction

Traditional Medicine (TM) has been for a longtime an important source of health care for people around the world [1–5]. This medicine includes diverse health practices, approaches, knowledge, and beliefs incorporating plant, animal, and/or mineral based medicines, spiritual therapies, manual techniques, and exercises applied singularly or in combination to maintain well-being, as well as to treat, diagnose or prevent illness [6]. Increasingly, in a growing number of African countries, traditional healers are trying to combine TM with conventional medicine (CM) practices and prefer to qualify themselves as being “*tradi-modern*” [7]. For the African population, Traditional African Medicine (TAM) is only effective for diagnosis and treatment of some diseases [8]. In TAM, diseases’ origin may be attributed to witches or spirits; this explains the special importance that Africans attach to it [9, 10]. In sub-Saharan Africa, the rising costs for conventional medical care [11], the presence of dysfunctional health systems, and an uneven distribution of structures and practitioners of conventional medicine, but also perceptions of illness, beliefs, and cultural influences concur to motivate recourse to traditional practitioners [12, 13].

In DR Congo, 40% of funding related to healthcare would weigh on the economy of the population, due to major dysfunctions of the existing health system [14]; more than 40% of hospitals are held by private actors, motivated mostly by their income [14, 15]. Data are quite difficult to collect in DR Congo, but (*i*) a 2009 study by the United Nations Development Program (UNDP) in Katanga indicated 2009 that 69.1% of this entity, the population lived in poverty, and their few incomes were largely (64.2%) spent on food [16]; (*ii*) the *Institut National de Statistique* (INS), indicated in 2012 that 61.2% of Congolese lived in rural areas with 60% of households living in extreme poverty [17]. The Ministry of Health regularly acknowledges too few and poorly distributed conventional generalists and specialists, depending on the region, the number of medical doctor ranges from 3.6 to 10 per 100,000 inhabitants [14]. Finally, both the World Bank [18] and a study by Wembonyama et al. [19] show low rates of use of conventional medical services.

By contrast, practically no study has investigated so far the repartition of traditional healers in DR Congo or even in Sub-Saharan Africa Given the proportions of TM recourse in those regions [20], tradipraticians are quite numerous, but also accessible throughout the territories, representing often the only source of health care for populations, both in rural and urban areas, regardless of the tribe or ethnic group [21–23]; which makes TM an important and accessible source of health care for all strata of the population [24]. For some 20 years now, the WHO has recognized the important contributions of traditional and complementary medicines to health care all over the world and has developed a voluntary policy to bring member states to integrate the locally important practices into their national health systems [25].

DR Congo is one of the WHO’s African Member States [6] and participates in the decisions taken to promote TAM through its integration into the Official Health System [26]. The Ministry of Health mentions the need for scientific investigations to understand different aspects of practice and use of this medicine, to successfully integrate it into the Official Health System [27, 28]. Moreover, several ethnobotanical surveys have shown the richness of natural resources used in the treatment of various diseases in TAM. In addition to encouraging results from chemical and biological screening of used species [29–37], recent studies continue to reveal the pharmacological properties of plant species from different regions of DR Congo [38–40].

Despite DR Congo implication in this policy, with a strong political will towards the development of integrative medicine, the local contexts of recourse to TM and the profiles and motivations of TM patients have not been elucidated. This information is however an important pre-requisite to the development and implementation of policies and regulations adapted to a rational and sustainable integrative medicine [27, 41]. An assertion of the WHO, according to which 80% of the African and Asian populations recourse to TM, constitutes the reference of several researchers, official reports, and the press [42], but this would be an unsubstantiated personal estimate dating back to 1983 [43]. This shows that each country should rely on data from well-conducted local studies. A bibliographical search was conducted in ScienceDirect, Google scholar, and PubMed (with keywords “Traditional medicine use”, “Herbal medicine use”, “DR Congo”; combined with AND); and indicated that the different surveys that determined the proportion of TM recourse in several DR Congo regions (Table 1) have not so far reported the local perceptions of TM, motivations for recourse to TM or the eventual fears towards this medicine. Additionally, the perception and use of traditional medicine can be influenced by certain parameters, such as educational level, financial means, religious belief, gender, cultural influence, marital status, etc. [25, 44–47]. Indeed, people with high education levels and/or the means to pay for conventional medicine care, could have a different perception of traditional medicine that possibly biases their health choice [48]. In addition, some religions may be reluctant towards TM, considering it a sin, or, on the contrary, may be adept of a particular care form [49, 50]. It thus appeared important to study the impact that such factors could have on the use of TM in DR Congo. The present study was carried out to evaluate and characterize the perceptions of TAM in the cosmopolitan city of Lubumbashi, the second city of DR Congo and capital of the Haut-Katanga province, to provide data for the guidance of researchers, political leaders, and practitioners of traditional and conventional medicines.

**Table 1:** Studies conducted in different categories of the population across DR Congo to determine the proportions of traditional medicine (TM) use.

Population categories	Study area	Key variable of interest	Sample size	Proportion of TM user (%)	Mode of TM use	Year	Ref
Survivors of the 1995 Ebola outbreak	Kikwit	Health experience during the Ebola illness	34	26	Not determined	1998	[51]
Inhabitants of the Kisenso municipality	Kinshasa	Perception of traditional practitioners by the populations	435 ^a^	100	Consultation of traditional practitioners	2013	[52]
Students 18–35 years old met on the campus of the University of Lubumbashi	Lubumbashi (Kassapa Campus)	Prevalence and characteristics of self-medication	515	84.9	Self-medication	2014	[53]
Pregnant women consulting health centers for antenatal consultation	Kipushi	Use of TM products during pregnancy	400	7.5	Not determined	2014	[54]
Hypertensive patients aged > 18 years resorting to primary health care	Kinshasa	Prevalence and determinant of traditional medicine use	280	26.1	Not determined	2014	[55]
Patients over 15 years of age interned in 10 HGR for malaria	Lubumbashi (hospitals)	Self-medication with malaria products	4960	73.6	Self-medication	2018	[56]
General population	Gbado-Lite	Knowledge and practices towards Covid-19	200	21.5 ^b^	Not determined	2020	[57]
Users of social networks.	Lubumbashi	Knowledge, attitudes, and practices towards Covid-19	478	52.8 ^b^	Self-medication	2020	[58]
Adults living in urban and rural areas	South Kivu	Prevalence and risk factors of chronic kidney disease	1317	22.2	Not determined	2020	[59]

^a^ A focus group of 15 people, supplemented by 35 focus groups of 12 people, in a sample of 5 districts

^b^ Proportion of people who have advocated the use of medicinal plants in case of symptoms associated with the coronavirus (Covid-19)

## Methodology

### Study area

**Fig 1:**
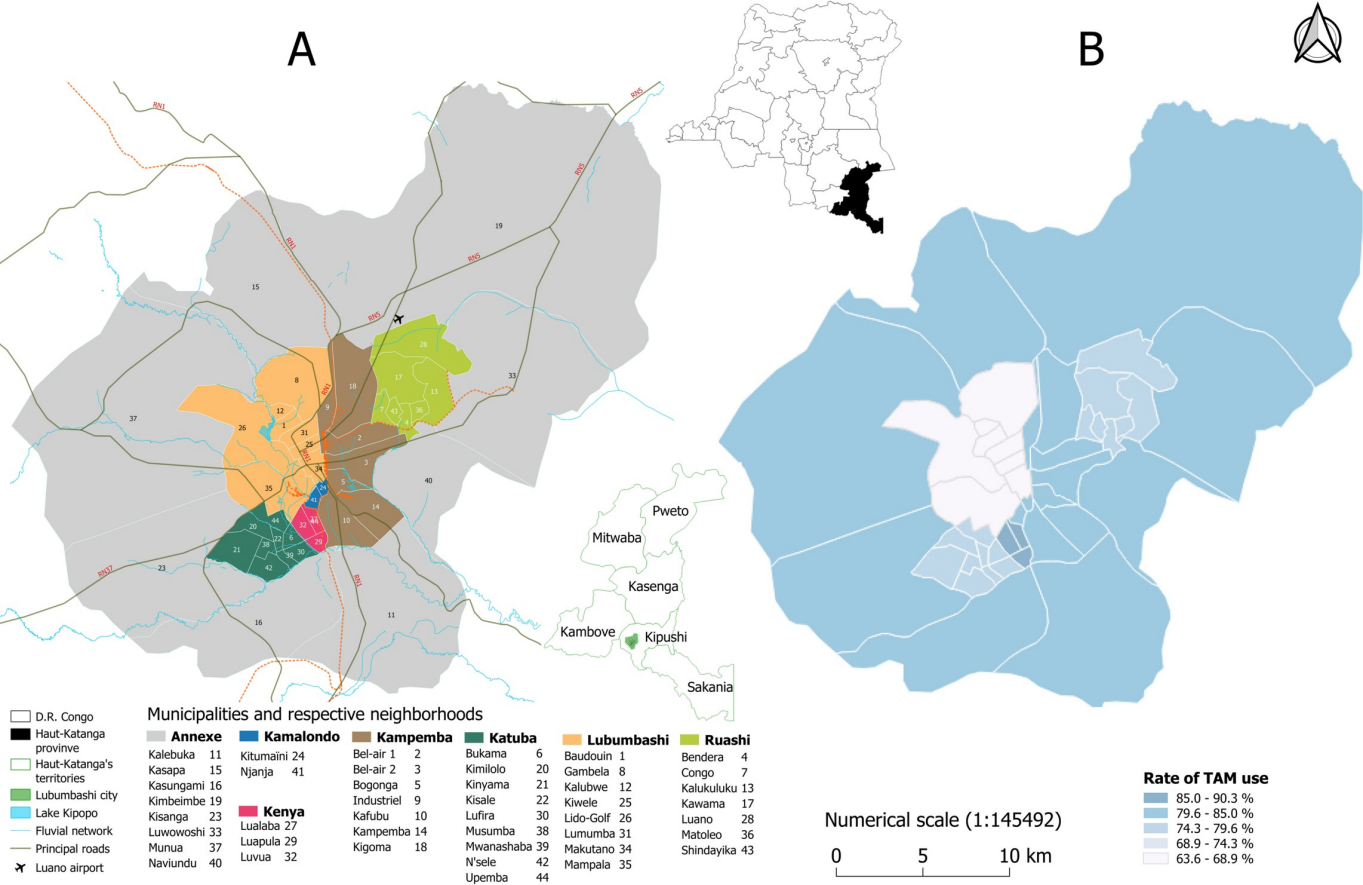
(A) Geographical map of Lubumbashi city and its situation in the Haut-Katanga province and in the Democratic Republic of Congo, (B) Choropleth map showing the levels of the TAM use in the different municipalities of Lubumbashi city. This map was created by the first author with the QGis 3.12 Open-Source software from shapefiles provided by the University of Lubumbashi Department of Geography.

Surveys have been conducted in the city of Lubumbashi (Fig 1), the capital of the Haut-Katanga province and covers an area of 747 km^2^ [60, 61]. Lubumbashi city is subdivided in 7 municipalities (communes), and 44 neighborhoods [62]. The city was created in 1910 during the Belgian colonization [63]. The expansion of mining and commercial activities made Lubumbashi an attractive city, leading to rural exodus and major displacements of inhabitants from other provinces to Lubumbashi [64]. This city comprises a very diverse population, coming from all over the country, and is characterized by a huge ethnic complexity [65, 66]. There has been no recent population census in DR Congo [17, 67, 68] but fragmentary data on inhabitants’ numbers and infrastructures are available at the *Institut National de Statistique* [69]. During the pre-colonial period, the population was probably exclusively treated with TAM. During colonization, CM was imposed as “the” official source of healthcare, especially in urban areas; TAM was even locally forbidden through churches that considered the practices as fetichism and sorcery. Nevertheless, attachment to tradition and especially belief that certain diseases are untreatable in CM, led the population to continue TAM practices [26]. The epidemiological profile of Lubumbashi is typical of tropical regions, characterized by the predominance of parasitic and infectious diseases [19, 70–75], but also includes some local diseases such as “*Kapopo*”, “*Nteta*”, “*Musanvu*”, *etc*., considered to be effectively cured in TAM [76–78]. Official healthcare structures are distributed within 11 Health zones [79]. These structures are mainly owned by private individuals whose primary interest lies in income, which leads to high variability in care prices [15, 80].

### Study design and sampling

This qualitative study was carried out from January to June 2017 and from January to July 2018. It adopted a two-stage sampling scheme [81], including stratified sampling [82] at the first stage, for the determination of the sample size and cluster sampling [83] at the second stage, for the selection of households to be included.

#### Sample size

To obtain representative samples, with an effective of interviewees sufficient to reduce error margins and improve data certainty [84, 85], the sampling scheme was drawn from the population estimates for each of the seven municipalities; these were provided, at the time of the survey, by the respective municipal offices (Table 2). Each municipality was considered as a stratum and representative sample sizes were determined online, using the calculator implemented on the CheckMarket website (https://fr.checkmarket.com/calculateur-taille-echantillon/). This calculator uses equation 1 (S3 File), developed by Cochran [86], to determine the size of the representative samples of large populations; and equation 2 (S3 File), to adjust the sample size according to the size of the studied population [87]. However, using the Cochran equation, the calculated sample size converges to around 384 respondents, for a mother population of more than 200,000 people, considering an error margin of 5% [88]. As the sizes of the populations of the different municipalities are significantly different, we opted to equilibrate the sampling across municipalities by sub-sampling according to the actual population numbers. The calculated minimal sample size of each municipality has exceeded of about 10 to 50% (Table 2), according to the corresponding population number and the availability and acceptance of people to participate in this study.

**Table 2:** Size of Lubumbashi’s municipalities populations and corresponding sample sizes.

Municipality	Investigation year	Population size^a^	Minimal representative sample (n_adj_)^b^	Surveyed people	Exceeding (%)^c^
Annexe	2018	399 632**	384	822	53.3
Lubumbashi	2018	323 062**	384	759	49.5
Katuba	2017	318 774*	384	661	41.9
Kampemba	2018	306 591**	384	660	41.8
Ruashi	2017	224 039*	384	497	22.7
Kenya	2018	112 283**	383	456	16.0
Kamalondo	2018	36 838**	381	423	9.9
Total			2679	4278	37.4

^a^ According to investigation year of a given municipality, we obtained population size for 2016*, or 2017**

^b^ Minimum sample size for population proportion of 0.50, error margin of 0.05 and confidence level of 0.95.

^c^ Percentages calculated on the number of surveyed people.

n_adj_: Adjusted sample size

The fact that the targeted people had the freedom to participate or not in the survey is probably the basis for the low proportion of women among the interviewees, given that women in our environment are less open to surveys as indicated by previous studies in which the liberty of respondents was guaranteed [29, 32]. However, the total number of interviewed women (1,562) is much higher than the minimum sample size (385) representative of women living in Lubumbashi city (about 2,000,000). Devising a study specifically targeted at women would be required for a comprehensive view of their opinions.

#### Ethical considerations

This study complies with the local and international ethical requirements, related to the survey conduction and the processing of data relating to interviewees. The study protocol was approved by the medical ethics committee of the Université de Lubumbashi, under number UNILU/CEM/087/2022, as part of the study project on the conditions for the traditional medicine integration into the official health system of DR Congo. The survey conduction and data presentation were carried out following the recommendations of the medical ethics committee of the University of Lubumbashi, the Declaration of Helsinki [89], as well as the European general regulations on personal data [90]. Before administering each questionnaire, each targeted person has explained the study, asked for free, and informed consent and invited to answer questions. Only people who accepted and gave their free and informed consent were included in this study. Consent was confirmed by signing the form prepared for this purpose (S4 File). Persons unable to sign were asked to affix their fingerprints using ink.

#### Informant selection and exclusion criteria

In each avenue of each municipality, we randomly choose a household and counts all fifth households to select the next. In the select households (considered as a cluster), the persons esteemed able to decide on the type of treatment to be used in case of illness (aged 20 and over), were asked to participate in this study. People under the age of 20 have been excluded from this study. People familiar with the investigators and/or this research project were also excluded to avoid bias.

### Data collection

Data were collected by a team of seven investigators, previously prepared for conducting semi-structured interviews and using a questionnaire guide containing 36 items (S1 File). The questionnaires have been administrated in face-to-face mode during a 60 to 90 minutes meeting with an informant. Most of the time respondent was met 2 times to carry out a whole interview. The survey successively questioned the informant on (*i*) socio-biographical data; (*ii*) whether she/he knows TAM and how she/he personally defines it; (*iii*) her/his eventual previous recourse to TAM and the diseases that were the causes for consultations; (*iv*) the motivations for recourse to TAM, the frequencies of consultations in TAM and CM, the treatment costs and payment methods; (*v*) her/his assessment of TAM efficacy; (*vi*) her/his criteria for selecting traditional caregivers; and (*vii*) her/his eventual fears about TAM.

### Statistical analysis

Univariate descriptive analyzes were used to calculate the citation frequency of qualitative variables’ modalities and present them in the form of graphs or frequency tables [91]. Diseases have been divided into different categories according to the International Classification of Diseases. For each variable, the missing values were excluded in both descriptive and static analyses. The Z test was used to compare the averages of age and monthly incomee among TAM user and non-users, given that the numbers in each group were greater than 30 [92]. A Chi-square tests was used to compare the percentages of people using TAM in the total sample and each municipality [6, 93, 94]. This test has also been used to study correlations between qualitative variables [95]. Where some observed numbers were less than 5, the exact Fischer test was used, since the Chi-square test is inapplicable in this case. Confidence intervals for the percentages of TAM use were calculated with equation 3 (S3 File). The significance and confidence level were fixed respectively at 5 and 95%. Statistical processing has been performed with the trial version of XLSTAT 2019.

## Results

### Surveyed population

This survey allowed us to meet 4278 people, including 36.5% of women (Table 3). The average age of the respondent was 32.1 ± 10.4 years, ranging from 20 to 84 years. Marital status is predominated by married people, and level of education by people with secondary education (Table 3). Minority declared having an occupation or profession at the survey time, and commercial activities were the most cited (Table 3). Only 28.4% quoted the amount of their monthly income (average = 174,1 ± 5.7 USD) at the survey time. The average monthly income of those who recourse to TAM (162.9 ± 6.4 USD) is statistically (*p* = 0.0002) lower than that of those who do not (213.1 ± 11.9 USD); a lower monthly income seems to contribute to the decision of TM recourse. Most said they lived in peri-urban areas; the ethnic groups were predominated by the Luba of Katanga and the religion, by the protestant Christians (Table 3).

**Table 3:** Sociobiographical data of interviewees.

Variables	Modalities	n	%	The proportion of TAM users % [95% IC]	Khi-square
Gender	Male	2716	63.5	77.1 [75.6–78.7]	22.956
	Female	1562	36.5	83.3 [81.4–85.1]	*p* < 0.0001 *
Age (years)	20 to 35	3024	70.7	78.2 [76.8–79.7]	
	36 to 50	1022	23.9	81.5 [79.1–83.9]	32.890
	51 to 65	170	3.9	82.9 [77.3–88.6]	*p* < 0.0001 *
	66 to 80	58	1.4	89.6 [81.8–97.5]	r = 0.9534 ^a^
	81 and over	4	0.09	100 [100–100]	
Education level	No studies	448	10.5	79.2 [75.5–82.9]	30.650
	Primary	269	6.3	88.5 [84.7–92.3]	*p* < 0.0001 *
	Secondary	1915	44.8	81.8 [80.0–83.5]	
	University	1632	38.1	75.3 [73.2–77.4]	
	No answer	14	0.3	57.1 [31.2–83.1]	
Profession or occupation at the survey time	No occupation	1166	27.3	81.2 [79.0–83.5]	7.142
	No answer	736	17.2	81.4 [78.6–84.2]	*p* = 0.308 ns
	Students	532	12.4	74.4 [70.7–78.1]	
	Commercial activities	505	11.8	82.4 [79.0–85.7]	
	Teachers	319	7.5	72.4 [67.5–77.3]	
	Farmers	169	3.9	71.6 [64.8–78.4]	
	Other	851	19.9	80.6 [78.0–83.3]	
Monthly income at the survey time ^b^	No answer	2524	58.9	78.7 [77.1–80.3]	23.118
	No precision	355	8.3	91.0 [88.0–94.0]	*p* = 0.001 *
	It’s a personal secret	165	3.9	71.5 [64.6–78.4]	r = - 0.8816 ^c^
	Less than 50 USD	207	4.8	86.5 [81.8–91.1]	
	50 to 150 USD	550	12.9	81.3 [78.0–84.5]	
	151 to 300 USD	324	7.6	72.5 [67.7–77.4]	
	More than 300 USD	153	3.6	69.9 [62.7–77.2]	
Marital statuts	Single	1831	42.8	74.1 [72.1–76.1]	15.048
	Married	2387	55.8	83.5 [81.9–84.9]	*p* = 0.002 *
	Divorced	11	0.3	63.6 [55.2–72.0]	
	Widowers	49	1.1	83.7 [73.3–94.0]	
Residential environment	Urban	1861	43.5	78.1 [76.3–80.0]	3.159
	Peri-urban	2417	56.5	80.3 [78.8–81.9]	*p* = 0.076 ns
Ethnic groups or tribes	Luba-Katanga	1094	25.6	80.7 [78.4–83.1]	8.857
	Luba-Kasai	604	14.1	79.6 [76.4–82.8]	*p* = 0.115 ns
	Lunda	322	7.5	78.6 [74.1–83.1]	
	Bemba	318	7.4	85.2 [81.3–89.1]	
	Other	1388	32.4	81.2 [79.1–83.3]	
	No answer	552	12.9	69.0 [65.2–72.9]	
Religion	Protestant Christians	2347	54.9	80.4 [78.8–82.0]	32.597
	Catholic Christians	1088	25.4	73.3 [70.7–76.0]	*p* < 0.0001 *
	Muslims	152	3.6	82.3 [76.2–88.3]	
	Kimbanguists	117	2.7	92.3 [87.5–97.1]	
	No answer	339	7.9	74.0 [69.4–78.7]	
	Other	235	5.5	[93.7–98.6]	

* Statistically significant correlation; ns: statistically insignificant correlation

^a^ Pearson’s correlation coefficient between the means of age ranges and the proportion of the TAM recourse

^b^ Monthly incomes quoted in Congolese Francs (CDF) have been converted to United State Dollars (USD) at the exchange rate of 1600 CDF for 1 USD (in august 2018)

^c^ Pearson’s correlation coefficient between the averages of monthly income ranges and the proportion of the TAM recourse.

### Knowledge of traditional medicine and recourse to the traditional healers

Among surveyed people (n = 4278), (*i*) 97.4% were familiar with the concept of TAM and 75.8% defined it as “*herbal treatment*”; the rest of informants either did not answer (9.6%) or defined TAM as “*ancestor medicine*” (6.9%), “*tradition based medicine*” (2.4%), “*natural products treatment*” (1.1%), or others concepts (6.6%); (*ii*) 79.4% acknowledged having recently recoursed to TAM, a proportion that more or less depends on their residential municipality, living environment (urban or suburban), study level, marital status and gender (Tables 3 and 4). The rate of TAM use in the municipality of Lubumbashi was significantly low compared to the total sample and all the other municipalities (Table 3).

**Table 4:** The proportion of TAM recourses according to the residential municipality of surveyed people in Lubumbashi.

Considered groups		Rate of TAM use (%) and 95% CI	Statistic comparison (Khi-square)						
			AN	KM	KP	KT	KN	LB	RS
Total sample (n = 4278)		79.4 [78.2–80.6%]	**ns**	*	*	**ns**	*	*	**ns**
Residential municipality	Annexe (n = 822)	82.5 [78.9–86.1%]	–	ns	ns	ns	ns	*	ns
	Kamalondo (n = 423)	84.1 [81.3–86.9%]	ns	–	ns	ns	ns	*	ns
	Kampemba (n = 660)	83.0 [80.3–85.7%]	ns	ns	–	ns	ns	*	ns
	Katuba (n = 661)	77.5 [74.6–80.3%]	ns	ns	ns	–	*	*	ns
	Kenya (n = 456)	90.3 [87.7–92.9%]	ns	ns	ns	*	–	*	*
	Lubumbashi (n = 759)	63.6 [60.0–67.3%]	*	*	*	*	*	–	*
	Ruashi (n = 497)	77.9 [74.0–81.7%]	ns	ns	ns	ns	*	*	–

ns: Insignificant difference

* Significant difference

AN: Annexe, KM: Kamalondo, KP: Kampemba, KT: Katuba, KN: Kenya, LB: Lubumbashi, RS: Ruashi

**Fig 2:**
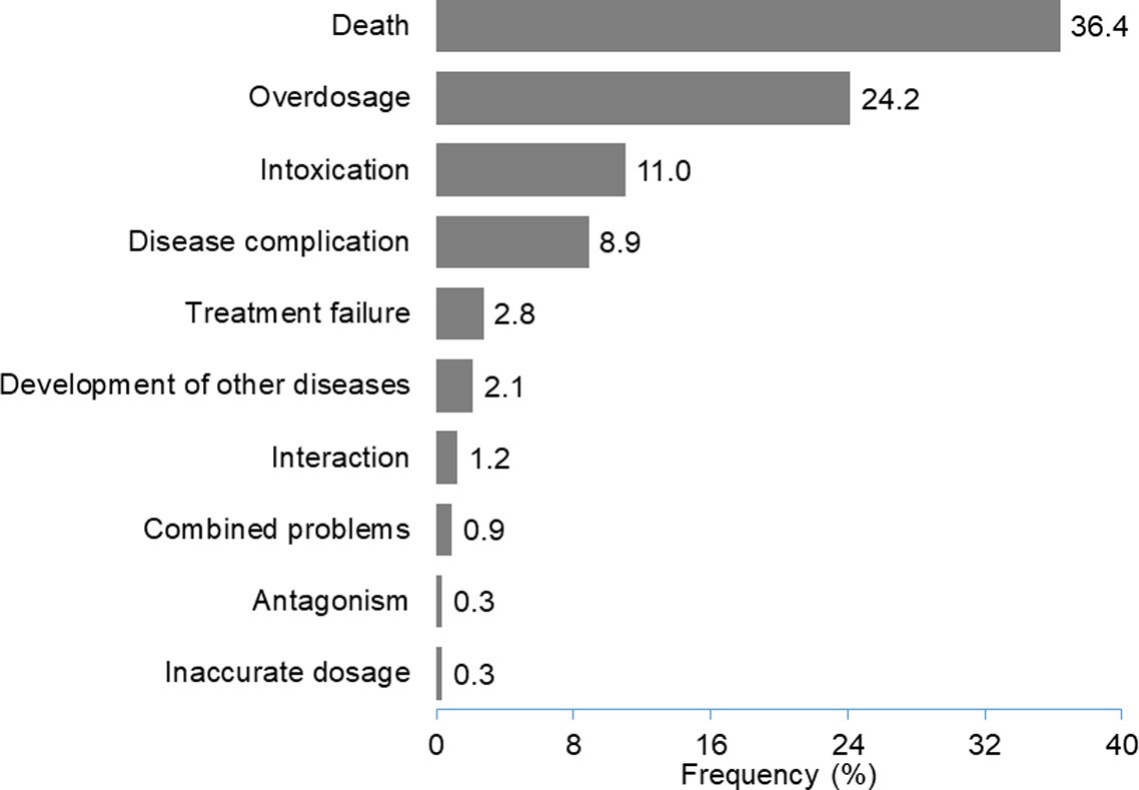
Risks quoted by those combining traditional and conventional medicine treatments (n = 327).

From the 3396 persons declaring that they use TM, (*i*) 30.4% had done it on their initiative, whereas 69.6% had followed a recommendation from friends (53.4%; n = 2363), family members (42.4%), conventional medicine doctors, and nurses (3.2%), or even from traditional healers (1%); (*ii*) most resorted to TM after conventional medicine failure (52.1%) while 34.7% directly started with TAM treatment and 13.2% had combined these two medicines. Among those who combined the two medicines, 36.4% (n = 448) were aware of possible risk, quoting death (42.1%), overdose (27.7%), intoxication (12.3%), and disease complication (10.2%) (Fig 2).

### Stated diseases and social problems for which TAM is resorted to

Traditional medicine has been resorted to for 164 “diseases” or symptoms grouped in 28 categories among which gastrointestinal (10.4%), veno-lymphatic (10.4%), gynecological (9.35%), and oral disorders (7.4%) are the most represented (S2 File). This list includes also symptoms recognized as non-specific in conventional medicine, and "local syndromes" (4.8%) as well as social problems (2.0%) (S2 File). These last categories include diseases and problems attributed to supernatural entities for which traditional medicine is considered the only care source. Individually, the most cited diseases include hemorrhoids (10.4%), dentary decay (7.3%), sterility (7.1%), malaria (4.4%), and abdominal pains (4.1%).

### Stated motivations for resorting to TAM

Several motivations were cited to explain the preference for traditional African medicine treatment. The most cited include especially efficacy, speed in healing, and low cost (Table 5).

**Table 5:** The major motivation for resorting to traditional African medicine in Lubumbashi city.

Cited motivations ^a^	(%) n = 3396
Efficiency	52.9
Speed of healing	19.1
Low cost	16.1
Specificity for certain diseases	2.3
Ease of use	2.3
Testimonials	1.8
Habit	1.4
Availability	1.1
No pain	0.8
Use of natural products	0.7
Confidentiality of traditional healers	0.4
Proximity	0.3
Protection against evil spirits	0.2
No surgical intervention	0.1
Others motivations ^b^	0.5

^a^ Interviewees were asked to cite the single most important reason motivating recourse to TAM

^b^ Some motivations whose quote frequency is less than à 0.1%

### Caregiver selection

**Fig 3:**
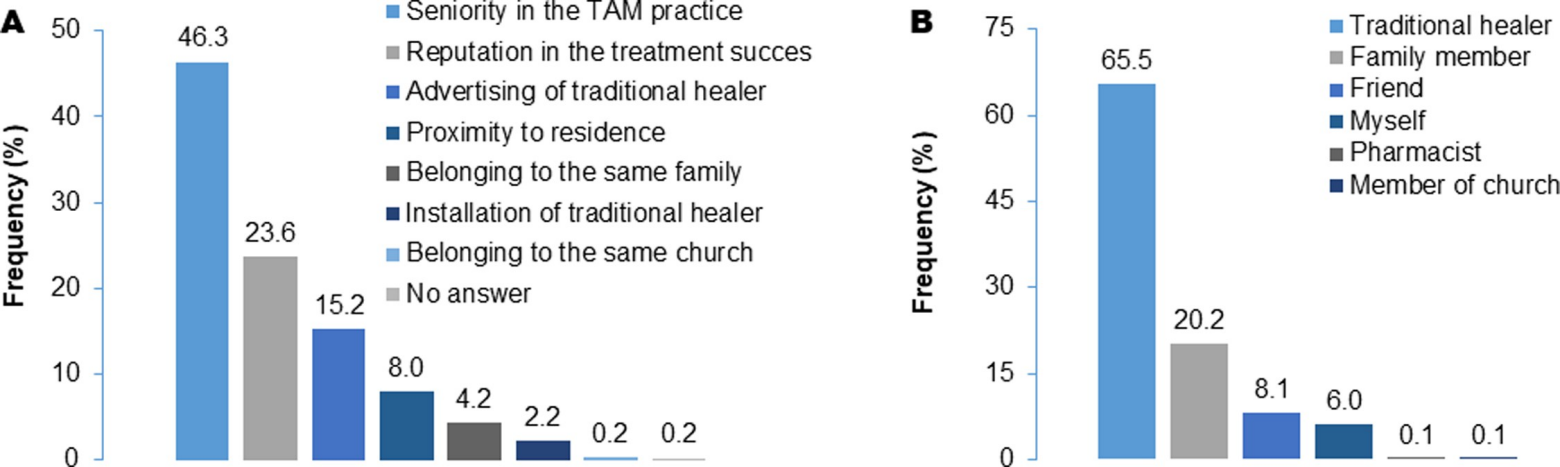
Applied criteria to choose caregivers (A) and categories of caregivers consulted by respondents who have recourse to traditional medicine (B).

A decision of TM recourse generally requires the selection of a given tradipratician among a vast choice of both generalists and specialists. Consulted caregivers (Fig 3) include traditional healers (65.5%, n = 3396), family members (20.2%), and friends (8.1%); some interviewees cited self-care, but their TAM knowledge level could not be verified. The main parameters guiding the selection of a caregiver include seniority (46.3%), the reputation of success (23.6%), advertising (15.2%), belonging to the patient’s family (8%), testimonials of patients, church membership, facilities, *etc*.

### Medication and administration routes

A total of 83.8% informants using TAM (n = 3396) answered about the nature of prescribed recipes. Most of them (76.4%) stated the presence of herbals in their medication whereas minerals, animals, and mixed products are much less frequent (7.4%); 16.2% used products described as “unknown”. These recipes have been administered enterally, by oral and anal routes, (43.7%) parenterally (scarification) (21.2%), topically (20.8%) (cutaneous, vaginal, urethral, ocular, or otic routes), or by a combination of routes (14.3%).

### Appreciation of traditional medicine treatments

The knowledge about safety and efficacy versus placebo is part of the elements required for integrating TM into an integrative health system. The stated effectiveness of TAM is so far mainly based on testimonials obtained from traditional healers and patients, their relatives, friends, and/or family members. From our informants who resorted to TM (n = 3396), 87% recognized that TAM was effective, while 11.6% testified the ineffectiveness of their treatment. Such indicators should be systematically sought for each combination of disease and treatment and evaluated for eventual convergence.

### Cost of healthcare in TAM

**Fig 4:**
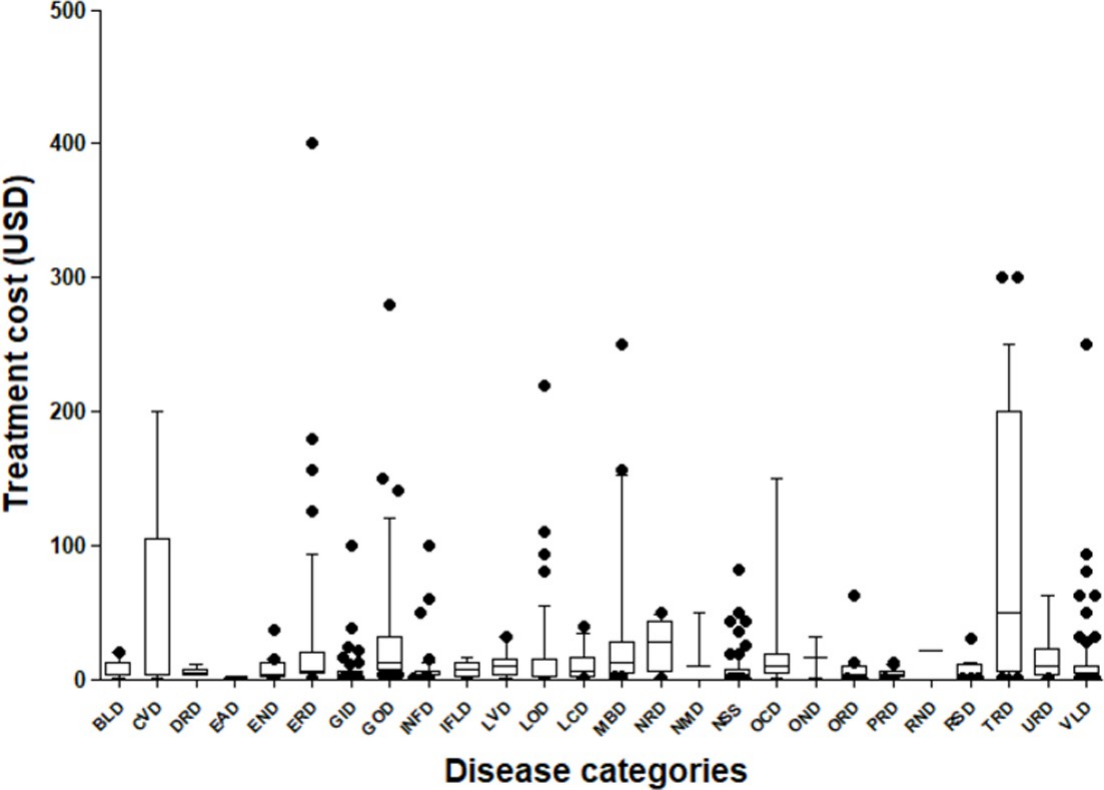
Variability of the treatment cost by diseases categories. The box corresponds to percentiles 25 and 75; the line to the median; the whiskers to percentiles 10 and 90. Abbreviations, BLD: Blood diseases; CVD: Cardiovascular diseases; DRD: Dermatological diseases; EAD: Eating disorders; END: Ear, Nose, and Throat Diseases; ERD: Erectile disorders; GID: Gastrointestinal disorders; GOD: Gyneco-obstetric disorders; INFD: Infectious diseases; IFLD: Inflammatory diseases; LVD: Liver problems; LOD: Local diseases; LCD: Locomotor disorders; MBD: Metabolic disorders; NRD: Neurological disorders; NMD: Neuromuscular disorders; NSS: Nonspecific symptoms; OCD: Ocular disease; OND: Oncological diseases; ORD: Oral disorders; PRD: Parasitic diseases; RND: Renal disorders; RSD: Respiratory disorders; TRD: Traumatic diseases, URD: Urological diseases, VLD: veino-capillary diseases.

Among those who resorted to TAM (n = 3396), 25.7% were treated for free, 71.5% paid in money or goods and 2.8% did not respond; reported cost of treatment corresponds to a modal of $ 3.12 (range, $ 0.03 to $ 400). Payments for traditional African medicine care are made after healing and are linked to patient satisfaction. Graph (Fig 4) shows interclass variability of treatment cost that is higher as the number of diseases is greater. Otherwise, exorbitant amounts have been noted for erectile disease and especially impotence. According to this result, TAM would be used by people of all ranks, as it offers care whose cost ranges from free to very high costs. In addition, high costs show that traditional medicine is not only used by people who do not have financial access to conventional medicine.

### Apprehensions and fears towards of TAM

Before undertaking any process of promotion and/or integration of TM in an integrative health system, the eventual apprehensions and/or fears towards TM are important to identify. From those who recourse to TM (n = 3396), 54.8% had no apprehensions and 6.2% refused to answer; the others quoted possible risks related to dose imprecision (8.6%), overdosage (8.6%), intoxication (6.6%), lack of hygiene (3.4%), prohibitions (2.4%) and performed ceremonials (4.6%) (Table 6). Among those who have not resorted to TAM (n = 882), 16,3% have no fears towards this medicine and 5.7% did not answer. Cited risks included dose imprecision (16%), witchcraft (13.7%), lack of hygiene (8.8%), intoxication (8%), and overdosage (7.6%) (Table 6). Statistical tests show that fears of dose inaccuracies and witchcraft are significantly predominant in the group of those who have not used TAM, while those who have no fear predominate among those who have resorted to this medicine (Table 6). In the TAM users’ group, apprehensions mainly relate to perceived weaknesses in traditional healer’s practices, i.e., risks related to the preparation and/or conservation of recipes, diagnosis, and healing ceremonies perceived as magical rituals. For the non-users of TAM, this medicine is more perceived as a practice reserved for the poorest patients (8.9%), related to witches and fetishism (21.5%).

**Table 6:** Fears for TAM, mentioned by informants who recoursed to traditional medicine and those who did not.

Cited fears	Those who have used TAM (%, n = 3396)	Those who did not use TAM (%, n = 882)	Chi-square (X²)
Bitter recipes	0.6	-	-
Rituals	2.0	-	-
Therapeutic failure	1.0	-	-
Fetishism	1.8	5.8	2.188 (*p* = 0.139)
Dose imprecisions	8.6	19.6	4.995 (*p* = 0.025) *
Intoxication	6.6	8.7	0.312 (*p* = 0.576)
It is shameful	1.4	4.4	1.598 (*p* = 0.206)
Lack of hygiene	3.4	8,8	2.545 (*p* = 0.111)
No fear	54.8	16.3	32.347 (*p* < 0,0001) *
Overdose	8.6	7.6	0.067 (*p* = 0.796)
Prohibitions	2.4	0.5	1.263 (*p* = 0.261)
Side effects	1.2	1.7	0.087 (*p* = 0.767)
Non trained healers	-	4.5	-
Witchcraft	0.5	15.7	15.519 (*p* < 0,0001) *
Worsening of the disease	0.9	0.7	0.025 (*p* = 0.874)
Non answer	6.2	5.7	*p* = 0.881)

* Statistically significant difference

### Prospects in case of failure of TAM treatment

People using TAM were asked about decisions they would make if treatment of TAM failed. Results show that most cited decisions include using conventional medicine (32.9%) and prayer (30.9%) as well as combining herbal treatment with Christian prayer (22.2%). Note that 4.8% believe that TAM treatment cannot fail and therefore do not foresee any decisions. The Chi-square test showed a significant correlation (*p* = 0.017), between the intended decision and study level. Decisions of informants with no education level and those at the primary and secondary levels essentially preferred to use prayer, while those at the university level prefer to use conventional medicine (Table 7).

**Table 7:** Decisions envisaged by TAM users in the event failure of their treatment.

Envisaged decisions	Frequencies (%) in all the TAM users (n = 3396)	Frequencies (%) according to the study level			
		No study (n = 448)	Elementary (n = 269)	Secondary (n = 1915)	University (n = 1632)
Change traditional healer	2.8	1.7	1.3	2.4	4.3
Continue treatment	1.4	0.6	2.1	0.8	2.1
Doubling dose	2.2	-	0.4	1.6	2.1
Mix the two medicines	1.4	2.8	0.8	1.3	1.3
Recourse to the Christian prayer	30.9	38.6	37.4	37.1	21.6
Repeat the treatment	2.1	4.2	3.4	1.7	-
Combine the TAM treatment and Christian prayer	21.5	19.5	24.8	20.4	23.2
Use conventional medicine	32.9	28.9	24.8	31.4	45.4
No decision (the treatment of TAM does not fail)	4.8	3.7	5.0	3.3	-

## Discussion

This study presents for the first time the perception of traditional medicine in Lubumbashi city, as well as the motivations and fears of patients and non-patients to this form of health care. However, the freedom of the people asked to participate in the survey induced a bias in the gender proportions, which predominated men. Despite the minority of women, their number in our sample (1,562) seems to compensate for this limit.

### Socio-biographical features of informants

Socio-biographical characteristics of people who participated in this study are important for understanding the profile of people resorting or not to TAM in Lubumbashi city. First, the type of people surveyed is predominantly male. This distribution is statistically different (*p* = 0.0124) from that of the general population revealed by the *Institut Nationale de Statistique* [17]. This can be explained by the freedom of the targeted informants to participate or not in this survey, meaning that most of men would have accepted it than women. However, the predominance of men among the people participating in different surveys conducted in Lubumbashi has been observed in several studies [29, 32, 33, 36, 37]. The Congolese population, like that of certain other regions of Africa, is predominantly young [17, 96]. The average age observed in this study 32.4 ± 10.5 years can be explained by the fact that people under the age of 20 were excluded from this study.

The professional situation of the informants was characterized by a remarkable rate of unemployed people. This corroborates the results of *Institut Nationale de Statistique* which highlights a job market failure in the urban areas of DR Congo [17, 97]. Furthermore, commercial activities, teaching, and agriculture which seem to predominate in the occupations of the respondents are also among the most practiced professions in DR Congo [17]. Despite this, the average income declared in this study is higher than the one (≈ 39 USD; 1USD = 1600 CDF) observed at the national level [17]. The average income observed in this study would be influenced by the fact that people with low incomes would be embarrassed to quote it, which can explain most non-responses to the question concerning the monthly income of respondents. The predominance of people living in peri-urban areas of the city of Lubumbashi can be explained by a strong increase in the population of this city and the new subdivisions in new districts not yet "urbanized" [64, 98, 99].

The proportions of marital status observed in this study are comparable to those of the population living in the Katanga region, found by the 1-2-3 survey in 2014 [17]. The Luba tribes (Luba-Kat and Luba-Kasaï), peoples of the same origin [66], predominate in this study because they are in the dominant tribes Lubumbashi city [65]. Indeed, the settlement of these tribes in the city of Lubumbashi began since the colonial period, with recruitments for the workforce of mining [100]. The predominance of Christians among the respondents would be explained on the one hand by their predominance in the Congolese population [101, 102] and on the other hand, by the fact that for a long time, the Congolese resort to TAM because of their strong belief in ancestors [50].

### Perception of traditional medicine by the population of Lubumbashi city

In the different Lubumbashi municipalities, the perceptions, and definitions of traditional medicine that we gathered indicate a good level of knowledge regarding its practices and practitioners. Indeed, 97.4% of surveyed people state to know TM while 79.4% recourse to this care system, indicating the importance of this medicine for people in DR Congo, as in most developing countries [6]. Traditional African medicine is mostly defined by the Lubumbashi population as "*herbals-based treatments*", a perception corresponding to the recipe’s composition they state, and common for traditional medicines all over the world [103]. The high rate of TAM recourse observed is close to those reported in Japan (76%) [104] and in Singapore (76%) [105] and may be explained by the urban/suburban settings of Lubumbashi with geographic accessibility to conventional medicine facilities. Nevertheless, such a massive recourse to TM requires the establishment and respect of appropriate regulations to control practices, practitioners, and products [106] but also the creation of a pharmacovigilance network to ensure the consumers’ safety [107–109]. We observed in this study as well as in others that the education level influences the rate of use of TAM [110, 111]. This situation would be explained by a certain difference in perception of TAM, which is sometimes influenced by the knowledge acquired at school [48]. Our comparison of TAM recourse according to income indicates that lower monthly incomes probably contribute to the choice of care. Nevertheless, the monthly income is overall quite low in Lubumbashi city as throughout the Katanga region where poverty was estimated at 69.1% in 2009 [16] and 66.6% in 2012 [17]. Poverty and economic accessibility to CM [17, 80] are probably among the major reasons for the massive recourse to TAM we recorded. This correlation is also observed in other African countries [112], for example in Tanzania [46].

In the present study, women recourse to TAM slightly more than men; this gender difference would be worth to be confirmed on a larger population sample to stratify according to age, marital status, and income. For Voeks [113], women show greater knowledge and attachment to TAM, compared to men This attachment would be favored by the "secret" nature of TM, allowing confidential management of pathologies related to femininity [114]; Shewamene et al. [112] showed that more than 80% (n = 11858) of African women recourse to TAM for maternal care. By contrast, Traore et al. [115], who worked in Burkina Faso CM care facilities, both in urban and rural areas, reported that encountered women indicate CM as a first therapeutic line for childbirth, childcare, and malarial fever; this choice was partly justified by the numerous awareness-raising campaigns directed to women.

There was no apparent influence of the living environment, tribal affiliation, or religion on the rate of TAM use, confirming its importance for Lubumbashi’s population, in line with data previously reported for Africa and, particularly, Congo [50]. Interestingly, these populations are reported to distinguish some diseases for which TM is the only possible care source [116]. However, in a study by Schioldann et al. [117], on the use of TM in the case of snake bites, the respondents stated that "*even if access to CM was improved*, *they would continue to use TM*, *because it’s part of the culture*". Upon a conventional medicine treatment failure, TM is mostly considered a last resort [118].

### Diseases and social problems stated to be treated by TAM

The importance of TAM still appears with a diversity of diseases and social problems for which respondents had consulted it. The presence of social problems among the reasons for consulting traditional healers shows that in Lubumbashi, TAM plays an important role that goes beyond simple medical care. This could be explained by the trust and social responsibility that the population places on traditional healers [119]. On the other hand, the presence of local diseases (not known or not managed in conventional medicine) shows the value and importance of TAM care for the population of Lubumbashi. The management of social problems and local diseases in TAM would be explained by the holistic character recognized in TAM [25, 120]. Some local diseases, particularly those affecting the gastrointestinal system (i.e., “*Lukunga*” and “*Kilonda tumbo*”), were again reported in Lubumbashi in 1994 [121]. According to the beliefs of local people, diseases caused by supernatural entities would find the best treatment in TAM [13, 122, 123] explaining why almost all consultations for these diseases are done in TAM. The predominance of hemorrhoids and dentary decay would be explained by the fact that these are chronic diseases for which TM is generally known to have the best treatments [25, 124, 125]. The frequency of malaria can be explained by its marked endemicity both in the city of Lubumbashi [73] and in other DR Congo regions [19]. Malaria is the reason for more consultations in CM [73] than among traditional healers of Lubumbashi [30, 37]. The representativeness of sterility among the reasons for using TAM can be explained by the fact that diseases affecting fertility, are often attributed to supernatural forces [76, 126].

### Grounds for the use of traditional medicine

Beyond a marked cultural influence on the recourse to TM, objective reasons were stated by the Lubumbashi population, notably TM effectiveness, speed of healing, and low cost. These patterns have also been observed in other studies in DR Congo, Kinshasa (effectiveness) [127], Tanzania (effectiveness and low cost) [46], Ivory Coast (effectiveness) [128], or Burma (effectiveness and low cost) [117]. TAM remains an indispensable tool; its integration into the health system can enable developing countries such as DR Congo to increase their health coverage and to globally improve their quality of healthcare [129]. Furthermore, its efficiency needs where possible to be proven by clinical studies for the maximum of known TAM products and practices [130]. As an example, a clinical study demonstrating the medicinal plant’s effectiveness in the treatment of AIDS [131], have been published and the results are favorable for TAM.

### Choice and type of caregivers consulted

Seniority, reputation for treatment success, advertising and family belonging are the main criteria stated for choosing a caregiver. The recognition of the traditional healer by his community [132, 133] and the success of his treatments would be major assets which are probably justified by the predominance of orality in TM practice and data sharing. However, consultation with people other than traditional healers, including friends, pharmacists, family, or church members, can be explained by a "*folk*" practice ("*popular*" practice) of TM widespread in Africa, i.e., knowledge and obtaining of remedies by non-specialists [132]. To some extent, such "*popular*" knowledge allows self-medication [134] as we observed in Lubumbashi city.

### Medications based on herbal medicines

Regarding medications, our study indicates 2 major points of attention. Medicinal plants, still a cornerstone for modern drugs’ discovery [103], are the main components of most TM treatments according to the interviewees (76.4%). A wide harvest of medicinal plants in nature, commonly practiced in *folk* medicine, may threaten valuable and important species, a situation likely to aggravate in the context of rapid population growth, like in DR Congo (3,5%/year) [68]. On the other hand, we noticed that some caregivers preserve the secret of their formula, hiding the nature and composition of dispensed products (9.8% of our interviewees know nothing about their remedy). This situation must be fought as it entails an unacceptable risk for the patient and prevents any pharmacovigilance measure.

### Cost of treatments

It is noteworthy that a high proportion (25.7%) of Lubumbashi patients benefited from free care; this is rarely the case in CM for which healthcare is becoming profitable merchandise in DR Congo [15, 80]. The costs (from very low to exorbitant) of treatments observed in this study confirm the economic accessibility of TAM but also that finance is not the first reason underlying a decision of TAM consulting.

### Apprehensions and fears towards TAM

Knowledge of the population’s apprehensions and fears towards TAM may help authorities in the planning of necessary actions. In Lubumbashi city, most TAM users (54.8%) feel no apprehension or fear when they resort to TAM. Such stated confidence in TAM is often explained by a feeling of patients that the practice is "*natural and innocuous*" [135, 136]. On the other hand, this confidence can be considered as a quite positive aspect, meaning that these interviewees have had no direct or indirect negative feedback of TAM. But this certainly depends on disease, diagnosis [137], practice, practitioner, and administered herbal products, which is worth investigating further. Most of the cited fears (Table 6) also require investigations that will lead to improvement of treatments; notably, the absence of standardization (of products, doses) and the poor presentation of TAM products induce apprehensions related to the dose that is administered (expressed as " *dose imprecision*", "*overdosage*", "*intoxication*") and the quality of preparations ("*lack of hygiene*", "*intoxication*").

The fears of those who do not resort to TAM indicate a need for actions likely to induce trust in practices and practitioners, in line with WHO recommendations [25]. These include an accreditation system for practitioners (define criteria), careful consideration of traditional diagnostic, practices (classify the acceptable practices, evaluate their efficacy, effectiveness, and risks), and products (hygiene, identity, and quality of herbals, standardization of the doses used, risks). All actions are required to build trust, promote, and progressively integrate TAM into the official health system.

The fears towards wizards and witch doctors are more immaterial; patients-linked, and they depend on his belief system and the practitioners he accepts or rejects. These considerations would come from some Christian churches [49] and the scientific lessons inherited from colonization [48, 138]. Christians would be the most embarrassed to maintain any relationship with caregivers resorting to supernatural forces because their church’s teaching is contrary to this [49, 139]. This sector, which seems impossible to regulate, should however not be neglected as it may play an important role in managing some mental disorders.

### Decisions made in case of TAM treatment failure

If TAM treatment fails, most informants would decide to use CM, prayer, or combine TAM treatment and prayer. The diversity of possibilities cited in this study shows that, in Lubumbashi, different means can be used in the search for health protection. Conventional medicine is therefore the most requested alternative. This shows that the organization of integrative health services, combining TAM and CM could not only optimize the use of healthcare services but also offer a service that integrates the needs of the maximum of patients. The use of prayer would be explained by considering evil supernatural forces as a possible source of certain illness and beneficial supernatural forces as a possible source of healing [21, 49, 140]. In Tanzania for example, Winkler et al. reported the recourse to the prayer for treatment of epilepsy which is believed to be a spiritually caused illness [141]. The recourse of traditional healers to invocations and prayers for the management of epilepsy has also been reported recently in Lubumbashi (DR Congo) by Koba et al. [142].

## Conclusion

Our study, conducted in the 7 municipalities of Lubumbashi city (DR Congo), indicates that TAM is a very popular practice and that people globally assimilate TAM to herbal treatments. The rate of recourse to this medicine appears very high (79.4%, 95% CI: 78.2–80.6), for diverse types of pathologies, notably hemorrhoids, dentary decay, malaria, and sterility as well as social problems, predominated by the bad luck. Recourse to TAM is mainly motivated by effectiveness, speed of healing, and low cost of treatments. This medicine was mainly consulted after conventional medicine treatment failure. Seniority, the reputation of treatment success, and advertising are the main factors invoked to select a caregiver. More than half of those who recourse to TAM are unafraid of the practice. For the others, and for those who do not recourse to TM, similar apprehensions and fears notably include dose imprecision, overdosage, intoxication, lack of hygiene, witchcraft, and fetishism. TAM appears as a popular and major source of healthcare for people living in Lubumbashi and is worth valorizing and regulating. Decision-makers must regulate, sanitize, and control this sector to ensure rational and safe use of this medicine. Quality, efficacy, and safety studies are essential to complete and enrich our knowledge, to fully understand the practices, and to sort out dangerous practices, practicians, and products to ensure the development of real integrative medicine to the benefit of DR Congo population.

## Supporting information

S1 File English interview protocol. (PDF)

S2 File Symptoms or diseases and social problems for which informants used traditional African medicine. (DOCX)

S3 File Used equations. (DOCX)

S4 File Free and informed consent form. (PDF)

S1 Table Definitions attributed to traditional African medicine. (XLSX)

S2 Table Cost of treatment according to disease categories. (XLSX)

## References

[1] Rivera JO, Loya AM, Ceballos R. Use of herbal medicines and implications for conventional drug therapy medical sciences. Altern Integr Med. 2013;2: 130. doi: 10.4172/2327-5162.1000130

[2] Murkherjee PK, Houghton P. The worldwide phenomenon of increased use of herbal products: opportunities and threats. Pharmaceut. In: Houghton P, Mukherjee PK, editors. Evaluation of Herbal Medicinal Products. Pharmaceut. London; 2009. pp. 3–12.

[3] Aslam MS, Ahmad MS. Worldwide importance of medicinal plants: current and historical perspectives. Recent Adv Biol Med. 2016;2: 88–93. doi: 10.18639/RABM.2016.02.338811

[4] Jamshidi-Kia F, Lorigooini Z, Amini-Khoei H. Medicinal plants: Past history and future perspective. J Herbmed Pharmacol. 2018;7: 1–7. doi: 10.15171/jhp.2018.01

[5] Panyod S, Ho C-T, Sheen L-Y. Dietary therapy and herbal medicine for COVID-19 prevention: A review and perspective. J Tradit Complement Med. 2020. doi: 10.1016/j.jtcme.2020.05.004 32691006 PMC7260602

[6] WHO. Traditional Medicine Strategy 2002–2005. World Health Organisation, WHO/EDM/TRM/2002.1, Geneva; 2002.

[7] Marsland R. The modern traditional healer: locating ‘hybridity’ in modern traditional medicine, southern Tanzania. J South Afr Stud. 2007;33: 751–765. doi: 10.1080/03057070701646845

[8] Truter I. African traditional healers: Cultural and religious beliefs intertwined in a holistic way. SA Pharm J. 2007;September: 56–60.

[9] Petrus ST, Bogopa DL. Natural and supernatural: Intersections between the spiritual and natural worlds in African witchcraft and healing with reference to southern Africa. Indo-Pacific J Phenomenol. 2007;7: 1–10.

[10] Florey MJ, Wolff XY. Incantation and herbal medicine: Alune ethnomedical knowledge in a context of changer. J Ethnobiol. 1998;18: 39–67.

[11] Deaton AS, Tortora R. People in sub-saharan Africa rate their health and health care among the lowest in the world. Health Aff. 2015;34: 519–527. doi: 10.1377/hlthaff.2014.0798 25715657 PMC5674528

[12] Bohoussou NS, Kone TB, Koffi BE. Typologie et motifs de recours aux soins de la médecine traditionnelle dans les quartiers Broukro, Koko, Houphouët-ville et Angouatanoukro (Bouaké). Regardsuds. 2017;2: 138–151.

[13] Gyasi RM, Asante F, Yeboah JYAW, Abass K, Mensah CM, Siaw LP. Pulled in or pushed out? Understanding the complexities of motivation for alternative therapies use in Ghana. Int J Qual Stud Health Well-being. 2016;11: 29667. doi: 10.3402/qhw.v11.29667 27018431 PMC4808739

[14] Berne-Wabern. Le système sanitaire à Kinshasa: médicaments et soins du VIH-sida, de l’hypertension artérielle, du diabète de type II et des troubles mentaux. Focus RD Congo: Confédération suisse, Office Fédéral des Migrations, Genève; 2014.

[15] Stasse S, Vita D, Kimfuta J, Campos V, Bossyns P, Criel B. Improving financial access to health care in the Kisantu district in the Democratic Republic of Congo: acting upon complexity. Glob Health Action. 2015;8: 25480. doi: 10.3402/gha.v8.25480 25563450 PMC4307026

[16] PNUD. Province du Katanga profil résumé de pauvrété et condition de vie des ménages. Programme des Nations Unies pour le Développement: Unité de lutte contre la pauvreté, Kinshasa; 2009.

[17] INS. Rapport global: Enquête 1-2-3. République Démocratique du Congo, Ministère du Plan et suivi de la mise oeuvre de la revolution de la modernité, Kinshasa; 2014.

[18] Mondiale Banque. Santé et pauvreté en République Démocratique du Congo: analyse et cadre stratégique de lutte contre la pauvreté, rapport d’état santé et pauvreté. Région Afrique, département du developpement humain, serie document du travail, Kinshasa; 2005.

[19] Wembonyama S, Mpaka L, Tshilolo L. Médecine et santé en République Démocratique du Congo: de l’indépendance à la 3e république. Médecie Trop. 2007;67: 447–457. 18225727

[20] James PB, Wardle J, Steel A, Adams J. Traditional, complementary and alternative medicine use in Sub-Saharan Africa: a systematic review. BMJ Glob Heal. 2018;3: e000895. doi: 10.1136/bmjgh-2018-000895 30483405 PMC6231111

[21] Mokgobi MG. Understanding traditional African healing. Afr J Phys Heal Educ Recreat Danc. 2015;20: 24–34. PMC4651463 26594664

[22] Puckree T, Mkhize M, Mgobhozi Z, Lin J. African traditional healers: what health care professionals need to know. Int J Rehabil Res. 2002;25: 247–251. doi: 10.1097/00004356-200212000-00001 12451299

[23] Zhang Q. Global situation and WHO strategy on traditional medicine. Tradit Med Mod Med. 2018;1: 11–13. doi: 10.1142/S257590001820001X

[24] Anyinam C. Availability, accessibility, acceptability, and adaptibility: Four attributes of African ethno-medicine. Soc Sci Med. 1987;25: 803–811. doi: 10.1016/0277-9536(87)90038-4 3317889

[25] WHO. Traditional medicine strategy 2014–2023. World Health Organization, Genva; 2013.

[26] Ministère de santé RDC. Politique et plan directeur de développement de la médecine traditionnelle. République Démocratique du Congo, Ministère de la santé publique, Kinshasa; 1999.

[27] LePhare. Médecine Traditionnelle: la RD Congo recense ses savants dans l’ombre. 2013 [cited 3 Nov 2019]. Available: http://new.lephareonline.net/medecine-traditionnelle-la-rd-congo-recense-ses-savants-de-lombre/

[28] Shengo LM, Mundongo TH. A survey of the antibacterial activity of three plants used in the Congolese herbal medicine practiced by the healers in the city of Lubumbashi: recent advancement. In: Chaitanya DMVNL, editor. Trends in Pharmaceutical Research and Development Vol 1. New York: Book Publisher International (a part of Sciencedomain International); 2020. pp. 81–91. doi: 10.9734/bpi/tprd/v1

[29] Bakari A, Mwamba M, Lumbu SJ-B, Duez P, Kahumba BJ. Ethnobotanical survey of herbs used in the management of diabetes mellitus in Southern Katanga Area/DR Congo. Pan Afr Med J. 2018;30: 1–13. doi: 10.11604/pamj.2018.30.218.11718 30574237 PMC6294981

[30] Kalonda ME, Mbayo M., Muhume KS, Kasereka M, Mulamba MJ, Manya MH, et al. Ethnopharmacological survey of plants used against malaria in Lubumbashi city (DR Congo). J Adv Bot Zool. 2014;1: 1–8. doi: 10.15297/JABZ.V1I2.02

[31] Okombe E V, Lumbu SJ-B, Stévigny C, Vandenput S, Pongombo SW, Duez P. Traditional plant-based remedies to control gastrointestinal disorders in livestock in the regions of Kamina and Kaniama (Katanga province, Democratic Republic of Congo). J Ethnopharmacol. 2014;153: 686–693. doi: 10.1016/j.jep.2014.03.027 24657601

[32] Kalunga M, Tshoto K, Cimanga CC, Mwamba M, Mbuyi KS, Kahumba BJ, et al. Survol ethnobotanique de quelques plantes utilisées contre la schistosomiase urogénitale à Lubumbashi et environs. Phytothérapie. 2014; 1–15. doi: 10.1007/s10298-014-0877-z

[33] Okombe E V, Pongombo SC, Duez P, Vandenput S. Remèdes vétérinaires traditionnels utilisés dans les élevages de chèvres à Lubumbashi et proche périphérie, RD Congo. Phytotherapie. 2014;12: 234–241. doi: 10.1007/s10298-014-0873-3

[34] Mbuyi KS, Kalunga M, Cimanga CC, Numbi wa I, Mbayo KM, Ali K, et al. Chemical screening of some reputed antimalarial plants used in Lubumbashi city and its surroundings. Phytochem BioSub J. 2018;12: 160–170.

[35] Kalonda ME, Mbayo KM, Kanangila BA, Muhume KS, Kahambu VZ, Tshisand P, et al. Evaluation of antisickling activity of some insect extracts from Katanga in Democratic Republic of the Congo. J Adv Med Life Siences. 2015;3: 1–5. doi: 10.15297/JALS.V3I1.04

[36] Bashige C V, Manya MH, Ntabaza N V, Numbi IE, Bakari AS, Kalonda ME, et al. Étude ethnobotanique, biologique et chimique de plantes réputées anticariogènes à Lubumbashi–RD Congo. Phytothérapie. 2015;15: 2–9. doi: 10.1007/s10298-015-1004-5

[37] Bashige C V, Bakari AS, Mbuyi K, Kahumba BJ, Duez P, Lumbu SJ-B. Etude ethnobotanique, phytochimique et évaluation de l’activité antiplasmodiale de 13 plantes réputées antipaludéennes dans la commune du Kenya (Lubumbashi, RDC). Phytothérapie. 2017. doi: 10.1007/s10298-017-1152-x

[38] Manya MH, Keymeulen F, Ngezahayo J, Bakari AS, Kalonda ME, Kahumba BJ, et al. Antimalarial herbal remedies of Bukavu and Uvira areas in DR Congo: An ethnobotanical survey. J Ethnopharmacol. 2020;249: 112422. doi: 10.1016/j.jep.2019.112422 31765762

[39] Mpiana PT, Ngbolua K, Tshibangu DST, Kilembe JT, Gbolo BZ, Mwanangombo DT, et al. Aloe vera (L.) Burm. F. as a Potential Anti-COVID-19 Plant: A Mini-review of Its Antiviral Activity. European J Med Plants. 2020;31: 86–93. doi: 10.9734/ejmp/2020/v31i830261

[40] Malimbo DK, Nyumu JK, Vitekere K, Mapoli J, Visando B, Mbumba J, et al. Exploitation of Pangolins (Pholidota Mammalia) by communities living in and around the Tayna nature reserve (RNT) North Kivu, Democratic Republic of Congo (DRC). J Geosci Environ Prot. 2020;08: 1–17. doi: 10.4236/gep.2020.84001

[41] Mposhi A, Manyeruke C, Hamauswa S. The importance of patenting Traditional Medicines in Africa: the case of Zimbabwe. Int J Humanit Soc Sci. 2013;3: 236–246. Available: http://www.ijhssnet.com/journals/Vol_3_No_2_Special_Issue_January_2013/26.pdf

[42] Oyebode O, Kandala N-B, Chilton PJ, Lilford RJ. Use of traditional medicine in middle-income countries: a WHO-SAGE study. Health Policy Plan. 2016;31: 984–991. doi: 10.1093/heapol/czw022 27033366 PMC5013777

[43] Africa Check. Do 80% of S. Africans regularly consult traditional healers? The claim is unproven. Rademeyer J, editor. 2013 [cited 6 Jun 2022]. Available: https://africacheck.org/fact-checks/reports/do-80-s-africans-regularly-consult-traditional-healers-claim-unproven

[44] Siti ZM, Tahir A, Farah AI, Fazlin SMA, Sondi S, Azman AH, et al. Use of traditional and complementary medicine in Malaysia: a baseline study. Complement Ther Med. 2009;17: 292–299. doi: 10.1016/j.ctim.2009.04.002 19942109

[45] Read SC, Carrier M-E, Whitley R, Gold I, Tulandi T, Zelkowitz P. Complementary and Alternative Medicine Use in Infertility: Cultural and Religious Influences in a Multicultural Canadian Setting. J Altern Complement Med. 2014;20: 686–692. doi: 10.1089/acm.2013.0329 25127071 PMC4155414

[46] Stanifer JW, Patel UD, Karia F, Thielman N, Maro V, Shimbi D, et al. The determinants of traditional medicine use in northern Tanzania: A mixed-methods study. PLoS One. 2015;10: e0122638. doi: 10.1371/journal.pone.0122638 25848762 PMC4388565

[47] Seo H-J, Baek S-M, Kim SG, Kim T-H, Choi SM. Prevalence of complementary and alternative medicine use in a community-based population in South Korea: A systematic review. Complement Ther Med. 2013;21: 260–271. doi: 10.1016/j.ctim.2013.03.001 23642959

[48] Zoure D. Collaboration entre médecine moderne médecine traditionnelle. Edition Universitaire Européenne; 2017.

[49] White P. The concept of diseases and health care in African traditional religion in Ghana. Teol Stud Stud. 2015;71: 1–7. doi: 10.4102/hts.v71i3.2762

[50] Mulemfo M. Traditional and Christian concepts of disease and healing among the Manianga. Teol Stud Stud. 1995;51: 338–357.

[51] De Roo A, Ado B, Rose B, Guimard Y, Fonck K, Colebunders R. Survey among survivors of the 1995 Ebola epidemic in Kikwit, Democratic Republic of Congo: their feelings and experiences. Trop Med Int Heal. 1998;3: 883–885. doi: 10.1046/j.1365-3156.1998.00322.x 9855400

[52] Manzambi KJ, Mbadu K V, Bruyère O, Reginster J-Y. Le tradipraticien est un acteur crédible dans l’offre des soins en territoire periurbain. Résultats d’une étude menée dans la commune de Kisenso à Kinshasa, Congo. In: Pacodel. Université de Liège, editor. Colloque Territoires périurbains: Développement, Enjeux et Perspectives dans les Pays du Sud. Gembloux; 2013. pp. 10–13.

[53] Bashige C V, Manya MH, Bakari AS, Sangwa KG, Kahumba BJ, Duez P, et al. Prévalence et caractéristiques de l’automédication chez les étudiants de 18 à 35 ans résidant au Campus de la Kasapa de l’Université de Lubumbashi. Pan Afr Med J. 2015;21: 1–12. doi: 10.11604/pamj.2015.21.107.5651 26327945 PMC4546724

[54] Khang IE, Kataka ZK, Kaki KM, Yayila NS, Wetshi Ongona TA, Lakula N, et al. Plants used by pregnant women at Kipushi city in Democratic Republic of Congo: prevalence and indications. Open Access Libr J. 2017;04: 1–8. doi: 10.4236/oalib.1103390

[55] Lulebo AM, Mapatano MA, Mutombo PB, Mafuta EM, Samba G, Coppieters Y. Prevalence and determinants of use of complementary and alternative medicine by hypertensive patients attending primary health care facilities in Kinshasa, Democratic Republic of the Congo: a cross-sectional study. BMC Complement Altern Med. 2017;17: 205. doi: 10.1186/s12906-017-1722-3 28390416 PMC5385009

[56] Bashige CV, Bakari AS, Okusa NP, Lumbu SJ-B. Self-medication with antimalarials drugs in Lubumbashi city (DR Congo). GSC Biol Pharm Sci. 2020;12: 7–20. doi: 10.30574/gscbps.2020.12.2.0228

[57] Falanga MC, Ndaba MM, Biye TNJ-C, Masengo AC, Basosila N, Iteku BJ, et al. Survey of COVID-19 knowledge by the population of Gbado-Lite city (Nord-Ubangi) in Democratic Republic of the Congo and new research perspectives. Eur J Sci Res. 2020;158: 157–166.

[58] Manya MH, Mutombo SC, Bashige CV, Nzuzi MG, Kabamba TA, Mutombo MA, et al. Knowledge, attitudes and practices among the population, towards COVID-19 in the Lubumbashi city (DR Congo): An online cross-sectional survey. World J Biol Pharm Heal Sci. 2021;05: 001–018. doi: 10.30574/wjbphs.2021.5.3.0016

[59] Masimango MI, Sumaili EK, Wallemacq P, Malembaka EB, Hermans MP, Fillée C, et al. Prevalence and risk factors of CKD in South Kivu, Democratic Republic of Congo: A large-scale population study. Kidney Int Reports. 2020;5: 1251–1260. doi: 10.1016/j.ekir.2020.05.028 32775824 PMC7403549

[60] Kasongo MP, Mukoko KG, Kipata ML, Lunda IJ-M. Elaboration de la carte géotechnique de la ville de Lubumbashi guide technique de sélection des sites d’implantation d’ouvrages du génie civil. Eur Sci J. 2018;14: 1857–7881. doi: 10.19044/esj.2018.v14n36p407

[61] Mutombo SC, Manya MH, Nsenga NS, Nzuzi MG, Kibwe MC, Maloba MJ, et al. Mineral elements analysis and total flavonoids content in the fresh leaves from two varieties of Hibiscus sabdariffa L. consumed as vegetable in Lubumbashi (DR Congo). World J Adv Res Rev. 2021;9: 147–155. doi: 10.30574/wjarr.2021.9.1.0512

[62] Mpinda MT, Abass OK, Bazirake MB, Nsokimieno EMM, Mylor NS, Kayembe KWM, et al. Towards the efficiency of municipal solid waste management in the Democratic Republic of Congo (DRC): Case Study of Lubumbashi. Am J Environ Sci. 2016;12: 193–205. doi: 10.3844/ajessp.2016.193.205

[63] Bustin E, Fetter B. The creation of Elisabethville, 1910–1940. Int J Afr Hist Stud. 1978;11: 561–565. doi: 10.2307/217339

[64] Mwembu D. La problématique de l’habitat dans la ville de Lubumbashi (Elisabethville), province du Katanga, 1910–1960. African Mi. In: Barker-Ciganikova M, Rüther K, Waldburger D, Bodenstein C-P, editors. The Politics of Housing in (Post-)Colonial Africa. African Mi. Boston: De Gruyter Oldenbourg; 2020. pp. 121–140. doi: 10.1515/9783110601183-005

[65] Mwembu D. L’épuration ethnique au Katanga et l’éthique du redressement des torts du passé. Can J Afr Stud. 2013;33: 483–499. doi: 10.1080/00083968.1999.10751170 19899233

[66] Hiernaux J. Luba du Katanga et Luba du Kasai (Congo); comparaison de deux populations de même origine. Bull Mem Soc Anthropol Paris. 1964;6: 611–622. doi: 10.3406/bmsap.1964.1292

[67] Nations Unies. Profils de pays 2017: République Démocratique du Congo. Commission économique pour l’Afrique Addis-Abeba; 2017. Available: https://www.uneca.org/fr/publications/profils-de-pays-2017

[68] Pourtier R. La République Démocratique du Congo face au défi démographique. Paris: Notes de l’Instut Français des Relations Internationales, Centre Afrique Subsaharienne; 2018.

[69] Ministère de Sante RDC. Plan National de Developpement Sanitaire 2016–2020: vers la couverture sanitaire universelle. République Démocratique du Congo, Ministère de la santé publique, Kinshasa; 2016. Available: http://www.nationalplanningcycles.org/sites/default/files/planning_cycle_repository/democratic_republic_of_congo/pnds_2016-2020_version_finale_29_avril_2016.pdf

[70] Crump JA. Typhoid fever and the challenge of nonmalaria febrile illness in sub-saharan Africa. Clin Infect Dis. 2012;54: 1107–1109. doi: 10.1093/cid/cis024 22357701

[71] Archibald LK, Reller BL. Clinical microbiology in developing countries. Emerg Infect Dis. 2001;7: 302–305. doi: 10.3201/eid0702.010232 11294729 PMC2631738

[72] Drain PK, Halperin DT, Hughes JP, Klausner JD, Bailey RC. Male circumcision, religion, and infectious diseases: An ecologic analysis of 118 developing countries. BMC Infect Dis. 2006;6: 172. doi: 10.1186/1471-2334-6-172 17137513 PMC1764746

[73] Mutombo AM, Mukomena E, Kanteng G, Kakisingi C, Mankan G, Mukuku OK, et al. Incidence of malaria in Lubumbashi, Democratic Republic of the Congo: An assessment of eight years. African J Heal Issues. 2018;2: 6–9. doi: 10.26875/ajhi232018xiii

[74] Moran A, Forouzanfar M, Sampson U, Chugh S, Feigin V, Mensah G. The epidemiology of cardiovascular diseases in Sub-Saharan Africa: The global burden of diseases, injuries and risk factors 2010 study. Prog Cardiovasc Dis. 2013;56: 234–239. doi: 10.1016/j.pcad.2013.09.019 24267430 PMC4031901

[75] Becker SL, Vogt J, Knopp S, Panning M, Warhurst DC, Polman K, et al. Persistent digestive disorders in the tropics: causative infectious pathogens and reference diagnostic tests. BMC Infect Dis. 2013;13: 37. doi: 10.1186/1471-2334-13-37 23347408 PMC3579720

[76] Kalau JM. Gestion domestique de la maladie et de la santé. In: Kakoma S, editor. Le profil sanitaire du Lushois. Observatoire du Changement Urbain. Lubumbashi; 2002. pp. 120–129.

[77] Adewuya AO, Makanjuola ROA. Lay beliefs regarding causes of mental illness in Nigeria: pattern and correlates. Soc Psychiatry Psychiatr Epidemiol. 2008;43: 336–341. doi: 10.1007/s00127-007-0305-x 18273532

[78] Makanjuola V, Esan Y, Oladeji B, Kola L, Appiah-Poku J, Harris B, et al. Explanatory model of psychosis: impact on perception of self-stigma by patients in three sub-saharan African cities. Soc Psychiatry Psychiatr Epidemiol. 2016;51: 1645–1654. doi: 10.1007/s00127-016-1274-8 27491966 PMC6311698

[79] SNIS. Rapport de distribution d’unités d’organisation. Ministère de la Santé, Système National d’Information Sanitaire, Province du Haut-Katanga, Lubumbashi; 2019.

[80] Ntambue AM, Malonga KF, Dramaix-Wilmet M, Ilunga TM, Musau AN, Matungulu CM, et al. Commercialization of obstetric and neonatal care in the Democratic Republic of the Congo: A study of the variability in user fees in Lubumbashi, 2014. PLoS One. 2018;13: e0205082. doi: 10.1371/journal.pone.0205082 30304060 PMC6179261

[81] Salehi MM, Smith DR. Two-stage sequential sampling: A neighborhood-free adaptive sampling procedure. J Agric Biol Environ Stat. 2005;10: 84–103. doi: 10.1198/108571105X28183

[82] Acharya AS, Prakash A, Nigam A. Sampling: why and how of it? Indian J Med Spec. 2013;4: 330–333. doi: 10.7713/ijms.2013.0032

[83] Thompson SK. Adaptive Cluster Sampling. J Am Stat Assoc. 1990;85: 1050–1059. doi: 10.1080/01621459.1990.10474975

[84] Paccagnella O. Sample size and accuracy of estimates in multilevel models. Methodology. 2011;7: 111–120. doi: 10.1027/1614-2241/a000029

[85] Maxwell SE, Kelley K, Rausch JR. Sample size planning for statistical power and accuracy in parameter estimation. Annu Rev Psychol. 2008;59: 537–563. doi: 10.1146/annurev.psych.59.103006.093735 17937603

[86] Cochran WG. Sampling techniques. 2nd ed. New York: Wiley & Sons, Inc; 1963.

[87] CheckMarket. La taille d’échantillon optimale. 2020 [cited 8 Jun 2020]. Available: https://fr.checkmarket.com/kb/comment-calculer-la-taille-d-echantillon/

[88] Israel GD. Determining sample size. Program Evaluation and Organizational Development, Florida Cooperative Extension Service, University of Florida, (Fact Sheet PEOD-6), Gainesville; 1992.

[89] World Medical Association. World Medical Association Declaration of Helsinki. JAMA. 2013;310: 2191–2194. doi: 10.1001/jama.2013.281053 24141714

[90] EU. General Data Protection Regulation. In: Official Journal of the European Union [Internet]. 2018 [cited 14 May 2020]. Available: https://gdpr-info.eu/art-32-gdpr/

[91] Krickeberg K, Van Trong P, Thi My Hanh P. Descriptive data analysis and statistics. 2nd ed. In: Krickeberg K, Trong P Van, Hanh PTM, editors. Statistics for biology and health, Epidemiology key to public health. 2nd ed. Springer Singapore; 2019. pp. 111–123. doi: 10.1007/978-3-030-16368-6_13

[92] Yadav SK, Singh S, Gupta R. Test of inference: One-sample or two-sample mean. In: Yadav SK, Singh S, Gupta R, editors. Biomedical statistics: A beginner’s guide. Springer Singapore; 2019. pp. 99–105. doi: 10.1007/978-981-32-9294-9_12

[93] Carlin J, Doyle L. Statistics for clinicians 5: Comparing proportions using the chi-squared test. J Paediatr Child Health. 2001;37: 392–394. doi: 10.1046/j.1440-1754.2001.00730.x 11532061

[94] Yadav SK, Singh S, Gupta R. Test for inference: categorical data II. In: Yadav SK, Singh S, Gupta R, editors. Biomedical Statistics. Springer Singapore; 2019. pp. 121–124. doi: 10.1007/978-981-32-9294-9_15

[95] Mirkin B. Eleven ways to look at the Chi-Squared coefficient for contingency tables. Am Stat. 2001;55: 111–120. doi: 10.1198/000313001750358428

[96] Pezzulo C, Hornby GM, Sorichetta A, Gaughan AE, Linard C, Bird TJ, et al. Sub-national mapping of population pyramids and dependency ratios in Africa and Asia. Sci Data. 2017;4: 170089. doi: 10.1038/sdata.2017.89 28722706 PMC5516541

[97] RDC. Analyse de contexte conjointe. 2015 [cited 13 Jul 2020] pp. 35–49. Available: https://cdn.vliruos.be/vliruos/667ee90e750440faf691bd10de2a9872.pdf

[98] Sikuzani YU, Boisson S, Kaleba SC, Khonde CN, Malaisse F, Halleux J-M, et al. Dynamique de l’ occupation du sol autour des sites miniers: analyse à long terme de la structure spatiale à Lubumbashi. Biotechnol Agron Soc Env. 2019;24: 14–27. doi: 10.25518/1780-4507.18306

[99] Useni SY, Cabala KS, Nkuku KC, Amisi MY, Malaisse F, Bogaert J, et al. Vingt-cinq ans de monitoring de la dynamique spatiale des espaces verts en réponse á l’urbanisation dans les communes de la ville de Lubumbashi (Haut-Katanga, R.D. Congo). Tropicultura. 2017;35: 300–311.

[100] Mushagalusa BA, Murhula BB, Mbangu MD. Yard farming in the city of Lubumbashi: Resident perceptions of home gardens in their community. J City Dev. 2019;1: 46–53. Available: https://www.researchgate.net/profile/Arsene_Balasha_Mushagalusa/publication/336459457_Yard_Farming_in_the_City_of_Lubumbashi_Resident_Perceptions_of_Home_Gardens_in_Their_Community/links/5da175fc45851553ff88ed41/Yard-Farming-in-the-City-of-Lubumbashi-Resi

[101] Target. Religion: les principales tendances en RDC révélées par une étude de Target. 2017 [cited 1 May 2020]. Available: https://www.target-sarl.cd/fr/content/religion-les-principales-tendances-en-rdc-revelees-par-une-etude-de-target

[102] La Croix Africa. Données géographiques et identités religieuses en République Démocratique du Congo. 2020 [cited 1 May 2020]. Available: https://africa.la-croix.com/statistiques/republique-democratique-congo/

[103] Karunamoorthi K, Jegajeevanram K, Vijayalakshmi J, Mengistie E. Traditional medicinal plants: A source of phytotherapeutic modality in resource-constrained health care settings. J Evid Based Complementary Altern Med. 2013;18: 67–74. doi: 10.1177/2156587212460241

[104] Yamashita H, Tsukayama H, Sugishita C. Popularity of complementary and alternative medicine in Japan: a telephone survey. Complement Ther Med. 2002;10: 84–93. doi: 10.1054/ctim.2002.0519 12481956

[105] Lim MK, Sadarangani P, Chan HL, Heng JY. Complementary and alternative medicine use in multiracial Singapore. Complement Ther Med. 2005;13: 16–24. doi: 10.1016/j.ctim.2004.11.002 15907674

[106] WHO. WHO global report on traditional and complementary medicine 2019. Geneva. Licence: CC BY-NC-SA3.0IGO; 2019.

[107] WHO. Pharmacovigilance for traditional medicine products: why and how? World Health Organization, Geneva; 2017.

[108] Williamson EM, Chan K, Xu Q, Nachtergael A, Bunel V, Zhang L, et al. Evaluating the safety of herbal medicines: Integrated toxicological approaches. Science (80-). 2015;347: 337–337. doi: 10.1126/science.347.6219.337-c

[109] Zhang L, Yan J, Liu X, Ye Z, Yang X, Meyboom R, et al. Pharmacovigilance practice and risk control of Traditional Chinese Medicine drugs in China: current status and future perspective. J Ethnopharmacol. 2012;140: 519–525. doi: 10.1016/j.jep.2012.01.058 22374080

[110] Thorsen RS, Pouliot M. Traditional medicine for the rich and knowledgeable: challenging assumptions about treatment-seeking behaviour in rural and. Health Policy Plan. 2016;31: 314–324. doi: 10.1093/heapol/czv060 26130610 PMC4779144

[111] Sarmiento I, Zuluaga G, Andersson N. Traditional medicine used in childbirth and for childhood diarrhoea in Nigeria’ s cross river state: interviews with traditional practitioners and a statewide cross-sectional study. BMJ Open. 2016;6: e010417. doi: 10.1136/bmjopen-2015-010417 27094939 PMC4838688

[112] Shewamene Z, Dune T, Smith CA. The use of traditional medicine in maternity care among African women in Africa and the diaspora: a systematic review. MC Complement Altern Med. 2017;17: 382. doi: 10.1186/s12906-017-1886-x 28768534 PMC5541739

[113] Voeks RA. Are women reservoirs of traditional plant knowledge? Gender, ethnobotany and globalization in northeast Brazil. Singap J Trop Geogr. 2007;28: 7–20. doi: 10.1111/j.1467-9493.2006.00273.x

[114] King R, Homsy J. Involving traditional healers in AIDS education and counselling in sub-Saharan Africa: a review. AIDS. 1997;11: S217–S225. 9451988

[115] Traore O, Ouedraogo A, Compaore M, Nikiema K, Zombre A, Kiendrebeogo M, et al. Social perceptions of malaria and diagnostic-driven malaria treatment in Burkina Faso. Heliyon. 2021;7: e05553. doi: 10.1016/j.heliyon.2020.e05553 33458436 PMC7797373

[116] Falisse J-B, Masino S, Ngenzebuhoro R. Indigenous medicine and biomedical health care in fragile settings: insights from Burundi. Health Policy Plan. 2018;33: 483–493. doi: 10.1093/heapol/czy002 29452365

[117] Schioldann E, Mahmood MA, Kyaw MM, Halliday D, Thwin KT, Chit NN, et al. Why snakebite patients in Myanmar seek traditional healers despite availability of biomedical care at hospitals? Community perspectives on reasons. PLoS Negl Trop Dis. 2018;12: e0006299. doi: 10.1371/journal.pntd.0006299 29489824 PMC5847227

[118] Antwi-baffour SS, Bello AI, Adjei DN, Mahmood SA, Ayeh-kumi PF. The place of traditional medicine in the African society: The science, acceptance and support. Am J Heal Res. 2014;2: 49–54. doi: 10.11648/j.ajhr.20140202.13

[119] Faye SL. Quand les tradithérapeutes ouest- africains soignent l’infertilité conjugale à Dakar (Sénégal): recompositions et dynamiques entrepreneuriales. Anthropol Santé. 2011;3: 1–21. doi: 10.4000/anthropologiesante.755

[120] CRDI. La médecine traditionnelle au Zaïre. Centre de Recherche pour le Développement International, Ottawa; 1979.

[121] Yoder PS. Examining ethnomedical diagnoses and treatment choices for diarrheal disorders in Lubumbashi Swahili. Med Anthropol. 1994;16: 211–247. doi: 10.1080/01459740.1994.9966116 8643023

[122] Kahissay MH, Fenta TG, Boon H. Beliefs and perception of ill-health causation: a socio-cultural qualitative study in rural North-Eastern Ethiopia. BMC Public Health. 2017;17: 124. doi: 10.1186/s12889-017-4052-y 28122606 PMC5267452

[123] Workneh T, Emirie G, Kaba M, Mekonnen Y, Kloos H. Perceptions of health and illness among the Konso people of southwestern Ethiopia: persistence and change. J Ethnobiol Ethnomed. 2018;14: 18. doi: 10.1186/s13002-018-0214-y 29482630 PMC6389056

[124] McCLure L, Flower A, Price S. Scoping the evidence for the effectiveness of herbal medicines. Eur Herb Tradit Med Pract Assoc. 2014; 1–46.

[125] Bordbar M, Pasalar M, Safaei S, Kamfirooz R, Zareifar S, Zekavat O, et al. Complementary and alternative medicine use in thalassemia patients in Shiraz, southern Iran: A cross-sectional study. J Tradit Complement Med. 2018;8: 141–146. doi: 10.1016/j.jtcme.2017.05.002 29322002 PMC5755989

[126] Tsobou R, Mapongmetsem PM, Van-Damme P. Medicinal plants used for treating reproductive health care problems in Cameroon, central Africa. Econ Bot. 2016;20: 1–15. doi: 10.1007/s12231-016-9344-0 27429475 PMC4927590

[127] Manzambi KJ, Mbadu K V, Balula SM-P, Mayamba KJ, Eloko EMG, Bruyère O, et al. Le role du tradipraticien dans l’offre des soins de sante de proximite en zones de sante semi-rurales: resultats d’une etude menee dans la commune peripherique de kisenso a kinshasa, congo. J d’Epidémiologie Santé Publique. 2014;13: 59–66.

[128] Kouakou EPY. Evaluation des motivations du choix de l’itinéraire thérapeutique des populations de Bouaké. Germivoir. 2015;2: 226–244.

[129] Skovgaard L, Nicolajsen PH, Pedersen E, Kant M, Fredrikson S, Verhoef M, et al. Use of complementary and alternative medicine among people with multiple sclerosis in the nordic countries. Autoimmune Dis. 2012;2012: 1–13. doi: 10.1155/2012/841085 23304461 PMC3529905

[130] Parveen A, Parveen B, Parveen R, Ahmad S. Challenges and guidelines for clinical trial of herbal drugs. J Pharm Bioallied Sci. 2015;7: 329. doi: 10.4103/0975-7406.168035 26681895 PMC4678978

[131] Tshibangu K, Worku Z, De Jongh M, Van Wyk A, Mokwena S, Peranovic V. Assessment of effectiveness of traditional herbal medicine in managing HIV/AIDS patients in South Africa. East Afr Med J. 2004;81: 499–504. doi: 10.4314/eamj.v81i10.9231 15715126

[132] Abayomi S. Plantes médicinales et médecine traditionnelle d’Afrique. 2nd ed. Karthala, editor. Karthala, Ibadan; 2010.

[133] Abubakar A, Van Baar A, Fischer R, Bomu G, Gona JK, Newton CR. Socio-cultural determinants of health-seeking behaviour on the Kenyan coast: a qualitative study. Jenkins N, editor. PLoS One. 2013;8: e71998. doi: 10.1371/journal.pone.0071998 24260094 PMC3832523

[134] Ekeke REC, Ekeopara CA. God, divinities and spirits in African traditional religious ontology. Am J Soc Manag Sci. 2010;1: 209–218. doi: 10.5251/ajsms.2010.1.2.209.218

[135] Moreira D de L, Teixeira SS, Monteiro MHD, De-Oliveira ACAX, Paumgartten FJR. Traditional use and safety of herbal medicines. Rev Bras Farmacogn. 2014;24: 248–257. doi: 10.1016/j.bjp.2014.03.006

[136] Zhang J, Onakpoya IJ, Posadzki P, Eddouks M. The safety of herbal medicine: from prejudice to evidence. Evidence-Based Complement Altern Med. 2015;2015: 1–3. doi: 10.1155/2015/316706 25838831 PMC4370194

[137] Afungchwi GM, Hesseling PB, Ladas EJ. The role of traditional healers in the diagnosis and management of Burkitt lymphoma in Cameroon: understanding the challenges and moving forward. BMC Complement Altern Med. 2017;17: 209. doi: 10.1186/s12906-017-1719-y 28399870 PMC5387296

[138] Cunningham AB. African medicinal plants: setting priorities in the interface of consravation and primary healthcare. Semple Alison, editor. People and Plants Initiative. Paris: UNESCO; 1993. Available: https://pdfs.semanticscholar.org/3404/adede6d135fd0a7ed30102ccbcf9013d7bce.pdf

[139] Lefèvre G. Les discours sur la médecine traditionnelle à Madagascar: Entre idéologie coloniale, salut de l’âme, raison économique, et pouvoir biomédical. Rev des Sci Soc. 2008; 46–59.

[140] Kahumba BJ, Rasamiravaka T, Okusa NP, Bakari AS, Bizumukama L, Kalonji NJ-B, et al. Traditional African medicine: From ancestral knowledge to a modern integrated future. Sciences (New York). 2015;350: S61–S63.

[141] Winkler AS, Mayer M, Ombay M, Mathias B, Schmutzhard E, Jilek-Aall L. Attitudes towards african traditional medicine and christian spiritual healing regarding treatment of epilepsy in a rural community of northern Tanzania. African J Tradit Complement Altern Med. 2010;7: 162–170. doi: 10.4314/ajtcam.v7i2.50877 21304629 PMC3021156

[142] Koba MC, Bakari A, Temfack ZL, Mugoli KR, Muteba KM, Koba BB. Knowledge, attitude and practice of traditional healers on epilepsy in Lubumbashi. Open Access Libr J. 2020;7: e6446. doi: 10.4236/oalib.1106446

