# Supplementary material for: Perceptions and use of traditional African medicine in Lubumbashi, Haut-Katanga province (DR Congo): A cross-sectional study
Source: PLoS One. 2022 Oct 18;17(10):e0276325. doi: 10.1371/journal.pone.0276325 (PMC9578634; doi:10.1371/journal.pone.0276325)
Supplement: S1 File — (PDF) [file pone.0276325.s001.pdf]

*Interview protocol*

**Investigation about the perception and use of traditional African medicine in Lubumbashi**

The main objective of this survey is to determine the percentage of the population of Lubumbashi that uses traditional medicine.

**Questionnaire**

1. Do you know traditional medicine? Yes ☐ No ☐
2. What is traditional medicine according to you :…………………………………………………………………
3. Have you ever resorted to traditional medicine? Yes ☐ ☐ No
   If so, for what disease and under what circumstances? :………………………………………………
4. Do you prefer traditional medicine? ☐ ☐ No
   What for? ……………………………………………………………………………………………………………
5. In which case you cannot resort to traditional medicine? :………………………………………………
6. How many times have you ever resorted to traditional medicine? :……………………………………..
7. Who did you get the care you needed (no name)? ……………………………………………………………
8. With which product did he care for you? :………………………………………………………………………
   How did you use it (route of administration):………………………………………………………………..
9. How did you choose your caregiver? :……………………………………………………………………………
10. How much was the cost of your treatment? :…………………………………………………………………
11. How did you identify (locate) your caregiver? :………………………………………………………………
12. What did you pay your bill with (caregiver fees) :……………………………………………………………
13. Have you ever treated yourself? : Yes☐ No☐
    With what recipe (drugs)? :……………………………………………………………………………………..
    For which disease? :…………………………………………………………………………………………………..
    How did you take your medicine? :………………………………………………………………………………
14. What fear do you have when resorting to traditional medicine? :……………………………………………
15. What fear do you have when resorting to modern medicine? ………………………………………………
16. 16. How did your caregiver discover your disease? :…………………………………………………………
17. At what precise point in the disease did you resort to traditional medicine: before, during or after resorting to modern medicine? :………………………………………………………………………………
18. Did you know that it is dangerous to mix the two treatments (traditional medicine / modern medicine)? Yes☐ No☐
19. What danger do you know exactly? :……………………………………………………………………………
20. Does your spouse agree to use traditional medicine? Yes☐ ☐ No

21. Your study level: No studies ☐ Primary ☐ Secondary ☐ Academic ☐
22. How many times have you resorted to modern medicine in last year? :…………………………………
23. For which disease did you resort to modern medicine in last year? : ………………………………………
24. Do you think traditional medicine is effective? Yes ☐ No ☐
25. Have you ever been recommended to use traditional medicine? : Yes ☐ No ☐
    If so, by whom?......................................................................................................................
26. Have you ever recommended someone to use Traditional Medicine? Yes ☐ No ☐
    If yes, who is it: A family member ☐ A friend ☐ A stranger ☐
27. What do you do in case of failure in modern medicine? :……………………………………………………..
28. What do you do in case of failure in traditional medicine? :……………………………………………………
29. Your residential address (district & commune):….......................................................................
30. Gender :………; Age :…………(Years); Marital status :…………………; Tribe:……………………………………
31. Your religion:………………………………………… Mother tongue:……………………………………………………
32. Languages spoken:…………………………………………………………………………………………………………..
33. Residence type: Urban ☐ Peri-urban ☐
34. What is your profession or occupation?...................................................................................……
35. How long have you lived in Lubumbashi? …………………………………………………………………………….
36. What is your monthly income? …………………………………………………………………………………………
37. What more can you add? ……………………………………………………………………………………………………
