# Supplementary material for: Perceptions and use of traditional African medicine in Lubumbashi, Haut-Katanga province (DR Congo): A cross-sectional study
Source: PLoS One. 2022 Oct 18;17(10):e0276325. doi: 10.1371/journal.pone.0276325 (PMC9578634; doi:10.1371/journal.pone.0276325)
Supplement: S2 File — (DOCX) [file pone.0276325.s002.docx]

**S2. File**

**Symptoms or diseases and social problems for which informants used traditional African medicine**

| **Disease categories and their sizes** | **Disease names** | **n** | **%** |
| --- | --- | --- | --- |
| Blood disorders (1.3%) | Anemia | 38 | 1.12 |
|  | Hemorrhage | 1 | 0.03 |
|  | Sickle cell disease | 5 | 0.15 |
| Cardiovascular disorders (0.94%) | Heart attack | 1 | 0.03 |
|  | Heart disease | 3 | 0.09 |
|  | High blood pressure | 25 | 0.74 |
|  | Hypotension | 3 | 0.09 |
| Dermatological disorders  (0.71%) | Black stain | 3 | 0.09 |
|  | Blister | 1 | 0.03 |
|  | Buttons in the face | 2 | 0.06 |
|  | Dermatosis | 2 | 0.06 |
|  | Hard buttons on the body | 1 | 0.03 |
|  | Shingling moth | 13 | 0.38 |
|  | Skin irritation | 1 | 0.03 |
|  | Tickling | 1 | 0.03 |
| Eating disorders  (0.15%) | Anorexia | 1 | 0.03 |
|  | Diet | 1 | 0.03 |
|  | kwashiorkor | 2 | 0.06 |
|  | Malnutrition | 1 | 0.03 |
| Ear, Nose, and Throat diseases  (4.80%) | Angina | 70 | 2.06 |
|  | Bleeding in the nose | 1 | 0.03 |
|  | Cold | 28 | 0.82 |
|  | Ear pain | 1 | 0.03 |
|  | Influenza | 4 | 0.12 |
|  | Mumps | 2 | 0.06 |
|  | Otitis | 10 | 0.29 |
|  | Pharyngitis | 1 | 0.03 |
|  | Sinusitis | 41 | 1.21 |
|  | Tonsillitis | 5 | 0.15 |
| Erectile disorders (3.59%) | Impotence | 24 | 0.71 |
|  | Loss of libido | 2 | 0.06 |
|  | Penis enlargement | 1 | 0.03 |
|  | Precious cumshot | 9 | 0.27 |
|  | Priapism | 1 | 0.03 |
|  | Sexual weakness | 85 | 2.50 |
| Gastrointestinal disorders (10.37%) | Abdominal pain | 140 | 4.12 |
|  | Anal pain | 1 | 0.03 |
|  | Appendicitis | 20 | 0.59 |
|  | Belly swelling | 5 | 0.15 |
|  | Bowel obstruction | 13 | 0.38 |
|  | Constipation | 6 | 0.18 |
|  | Diarrhea | 31 | 0.91 |
|  | Gastritis | 127 | 3.74 |
|  | Hiccough | 2 | 0.06 |
|  | Nausea | 5 | 0.15 |
|  | Sleeping sickness | 1 | 0.03 |
|  | Vomiting | 1 | 0.03 |
| Gynecological disorders (9.35%) | Agalactorrhoea | 2 | 0.06 |
|  | Childbirth | 4 | 0.12 |
|  | Dysmenorrhea | 30 | 0.88 |
|  | Dystocia | 1 | 0.03 |
|  | Facilitate childbirth | 6 | 0.18 |
|  | Frigidity | 9 | 0.27 |
|  | Gynecomastia | 1 | 0.03 |
|  | Menstruation disorder | 2 | 0.06 |
|  | Ovarian cyst | 11 | 0.32 |
|  | Polymenorrhea | 2 | 0.06 |
|  | Spontaneous abortion | 5 | 0.15 |
|  | Sterility | 242 | 7.13 |
|  | Swelling of the breasts | 1 | 0.03 |
|  | White loss | 1 | 0.03 |
| Immunological disorders | Allergy | 1 | 0.03 |
| Infectious diseases (8.13%) | Abscess | 8 | 0.24 |
|  | Boil | 10 | 0.29 |
|  | Buruli ulcer | 3 | 0.09 |
|  | Cholera | 1 | 0.03 |
|  | Dysentery | 6 | 0.18 |
|  | Gangrene gas | 1 | 0.03 |
|  | Genital herpes | 1 | 0.03 |
|  | Gonorrhea | 2 | 0.06 |
|  | HIV | 2 | 0.06 |
|  | Infection | 15 | 0.44 |
|  | Meadow | 2 | 0.06 |
|  | Measles | 9 | 0.27 |
|  | Meningitis | 1 | 0.03 |
|  | Mycosis | 14 | 0.41 |
|  | Scabies | 3 | 0.09 |
|  | Septicemia | 1 | 0.03 |
|  | Syphilis | 18 | 0.53 |
|  | Tuberculosis | 23 | 0.68 |
|  | Typhoid fever | 102 | 3.00 |
|  | Urinary infection | 36 | 1.06 |
|  | Vaginal infection | 1 | 0.03 |
|  | Varicella | 7 | 0.21 |
|  | Whitlow | 5 | 0.15 |
|  | Yellow fever | 5 | 0.15 |
| Inflammatory diseases (2.83%) | Inflammation | 1 | 0.03 |
|  | Prostatitis | 13 | 0.38 |
|  | Rheumatism | 75 | 2.21 |
|  | Splenitis | 7 | 0.21 |
| Liver problems (0.77%) | Hepatitis | 2 | 0.06 |
|  | Jaundice | 11 | 0.32 |
|  | Liver cirrhosis | 13 | 0.38 |
| Local syndromes  (4.8 %) | Kamondo | 2 | 0.06 |
|  | Kapopo | 50 | 1.47 |
|  | Kasumbi | 3 | 0.09 |
|  | Kilonda tumbo | 65 | 1.91 |
|  | Kunde | 2 | 0.06 |
|  | Lukunga | 6 | 0.18 |
|  | Mpese | 1 | 0.03 |
|  | Musamvu | 4 | 0.12 |
|  | Nsundu | 1 | 0.03 |
|  | Nteta | 29 | 0.85 |
| Social problems (2.0%) | Bad luck | 65 | 1.91 |
|  | Demonic possession | 2 | 0.60 |
|  | Love sickness | 1 | 0.03 |
| Locomotor disorders (2.83%) | Articular pain | 4 | 0.12 |
|  | Back pain | 37 | 1.09 |
|  | Bony pain | 1 | 0.03 |
|  | Edema of the foot | 2 | 0.06 |
|  | Foot pain | 1 | 0.03 |
|  | Low back pain | 12 | 0.35 |
|  | Lower back pain | 1 | 0.03 |
|  | Pain in the hip | 23 | 0.68 |
|  | Pain in the leg | 5 | 0.15 |
|  | Swelling of the feet | 5 | 0.15 |
|  | Swelling of the hand | 1 | 0.03 |
|  | Swelling of the legs | 4 | 0.12 |
| Metabolic disorders (3.24%) | Asthenia | 3 | 0.09 |
|  | Diabetes | 97 | 2.86 |
|  | Goitre | 6 | 0.18 |
|  | Hypercholesterolemia | 1 | 0.03 |
|  | Hyperuricemia | 2 | 0.06 |
|  | Overweight | 1 | 0.03 |
| Neurological disorders (2.3%) | Anxiety | 1 | 0.03 |
|  | Epilepsy | 35 | 1.03 |
|  | Fear of heights | 3 | 0.09 |
|  | Madness | 2 | 0.06 |
|  | Migraine | 6 | 0.18 |
|  | Nocturnal enuresis | 2 | 0.06 |
|  | Overwork | 24 | 0.71 |
|  | Paralysis | 5 | 0.15 |
| Neuromuscular disorders (0.29%) | Convulsion | 2 | 0.06 |
|  | Muscle pain | 8 | 0.24 |
| Nonspecific diseases (6.93%) | All diseases | 16 | 0.47 |
|  | Amulets | 2 | 0.06 |
|  | Disease prevention | 2 | 0.06 |
|  | Doubtful disease | 1 | 0.03 |
|  | Fever | 8 | 0.24 |
|  | Generalized malaise | 4 | 0.12 |
|  | Generalized pain | 1 | 0.03 |
|  | Headache | 36 | 1.06 |
|  | Pain in the chest | 1 | 0.03 |
|  | Poisoning | 8 | 0.24 |
|  | Several diseases | 156 | 4.59 |
| Ocular disease (0.85%) | Cataract | 2 | 0.06 |
|  | Conjunctivitis | 2 | 0.06 |
|  | Exophthalmos | 1 | 0.03 |
|  | Eye damage | 16 | 0.47 |
|  | Glaucoma | 5 | 0.15 |
|  | Stye | 2 | 0.06 |
|  | Vision disorder | 1 | 0.03 |
| Oncological disorders (0.65%) | Breast cancer | 5 | 0.15 |
|  | Cancer | 4 | 0.12 |
|  | Malignant tumor | 9 | 0.27 |
|  | Skin cancer | 3 | 0.09 |
|  | Tumor in the eye | 1 | 0.03 |
| Oral disorders (7.39%) | Bad breath | 2 | 0.06 |
|  | Tooth decay | 249 | 7.33 |
| Parasitic diseases (5.24%) | Amoebiasis | 3 | 0.09 |
|  | Elephantiasis | 1 | 0.03 |
|  | Intestinal worms | 20 | 0.59 |
|  | Malaria | 150 | 4.42 |
|  | Sleeping sickness | 4 | 0.12 |
| Renal disorders (0.27%) | Clogged kidney | 1 | 0.03 |
|  | Renal failure | 6 | 0.18 |
|  | Renal problems | 2 | 0.06 |
| Respiratory disorders (4.03%) | Asthma | 13 | 0.38 |
|  | Bronchitis | 2 | 0.06 |
|  | Cough | 120 | 3.53 |
|  | Dyspnea | 2 | 0.06 |
| Traumatic diseases (3.45%) | Bee sting | 2 | 0.06 |
|  | Burn | 14 | 0.41 |
|  | Dog bite | 4 | 0.12 |
|  | Fracture | 60 | 1.77 |
|  | Injury | 9 | 0.27 |
|  | Lightning | 1 | 0.03 |
|  | Monkey bite | 1 | 0.03 |
|  | Snake bite | 14 | 0.41 |
|  | Wound | 12 | 0.35 |
| Urological disorders | Hernia | 82 | 2.4 |
| Vein-lymphatic disorders (10.39%) | Hemorrhoid | 352 | 10.37 |
|  | Stretch marks | 1 | 0.03 |

**Disease category frequencies**

**Brief explanations of local diseases**

1. **Kamondo**: a disease that strikes children (under 6 years old) and characterized by fever, swelling of the tonsils and epiglottis, causing difficulty in swallowing and vomiting.
2. **Kapopo**: disease caused by people due to conflict and characterized by swelling and sore on the neck or cheek.
3. **Kasumbi**: it is an affection that strikes the child (under 3 years old) whose anus becomes reddish.
4. **Kilonda tumbo**: proctitis accompanied by prolonged diarrhea. Its symptoms are hyperemia of the anal margin and rectal prolapse (outflow of the rectal mucosa during the effort of defecation).
5. **Kunde**: disease characterized by tickling of the nipples and vagina, it can make a woman infertile or caused death to the children born to the suffering woman.
6. **Lukunga:** a kind of dehydration that often affects infants and is deemed incurable in hospitals. She has among her symptoms the depression of the fontanel of the child (under 5 years old).
7. **Mpese**: pathology considered to be thrown by a woman on another who had sex with her husband. This disease resembles shingles in its eruptions, but the vesicles are scattered all over the body, and the person who suffers from it feels pruritus.
8. **Musamvu**: disease of children (from 0 to 10 years old), which causes fever followed by convulsions and revulsion of the eyes. According to some THs, poor management of this disease exposes the person to epilepsy in adulthood.
9. **Nsundu**: disease characterized by the appearance of a cyst in the anus following an evolution of the hemorrhoid. The cyst would lead to weight loss for the patient and sterility for women.
10. **Nteta**: disease caused by people due to conflict; is characterized by swelling of the feet, is at the same time a localized ailment in the feet.
