# Supplementary material for: Perceptions and use of traditional African medicine in Lubumbashi, Haut-Katanga province (DR Congo): A cross-sectional study
Source: PLoS One. 2022 Oct 18;17(10):e0276325. doi: 10.1371/journal.pone.0276325 (PMC9578634; doi:10.1371/journal.pone.0276325)
Supplement: S3 File — (DOCX) [file pone.0276325.s003.docx]

**S3. File**

**Equations used**

***Sample size***

$n=\frac{\left( Z_{\alpha} \right)^{2}\cdot p\cdot\left( 1-p \right)}{ⅈ^{2}}$ (1) $n_{ad_{j}}=\frac{n}{\frac{n-1}{N}+1}$ (2)

With n = sample size; Z_α_ = 1.96 (Z value corresponding to the fixed error margin, that is 0.05 in this study); p = population proportion (assumed to be 0.5 since this would provide the maximum sample size) and *i* = error margin [1]; n_adj_ = adjusted sample size; N = population size [2].

***Confidence interval***

CI_0.95_ = $p\pm\left| z_{\alpha} \right|\sqrt{\frac{p\left( 1-p \right)}{n}}$ (3) [3–5]

With CI_0.95_: confidence interval at 95 %; *p*: observed percentage of people using TAM; Z_α_ : Z value corresponding to the fixed error margin; and n: used sample size.

***Cited references***

1. Israel GD. Determining sample size. Program Evaluation and Organizational Development, Florida Cooperative Extension Service, University of Florida, (Fact Sheet PEOD-6), Gainesville; 1992.

2. CheckMarket. La taille d’échantillon optimale. 2020 [cited 8 Jun 2020]. Available: https://fr.checkmarket.com/kb/comment-calculer-la-taille-d-echantillon/

3. Naing NN. Determination of sample size. Malaysian J Med Sci. 2013;10: 84–86. Available: https://europepmc.org/article/med/23386802

4. Agresti A, Caffo B. Simple and effective confidence intervals for proportions and differences of proportions result from adding two successes and two failures. Am Stat. 2000;54: 280–288. Available: http://www.jstor.org/stable/2685779

5. Jones V. Confidence intervals - One population mean and proportion testing. 3rd ed. In: Kendall Hunt, editor. Van Jones’ Conversational Statistics. 3rd ed. Dubuque; 2012. pp. 197–232.
