# Supplementary material for: Perceptions and use of traditional African medicine in Lubumbashi, Haut-Katanga province (DR Congo): A cross-sectional study
Source: PLoS One. 2022 Oct 18;17(10):e0276325. doi: 10.1371/journal.pone.0276325 (PMC9578634; doi:10.1371/journal.pone.0276325)
Supplement: S4 File — (PDF) [file pone.0276325.s004.pdf]

Survey on the perception and use of the Traditional African Medicine (TM) in Lubumbashi

Free and informed consent form

The study to be conducted is part of a doctoral research. It requires a survey to study conditions for the integration of Traditional Medicine into the official health system of DR Congo. To do this, your answers to questions related to TM and those relating to your person will be used to help determine the best conditions for integrating traditional medicine into the health official system.

All information collected will be kept confidential and encoded, the key to which will be held by the principal investigator; its will be processed and published anonymously.

By participating in this interview, you do not run any risk and, but you will not be paid for your participation. You participate freely in the survey, and at any time, you can stop your participation without having to provide an explanation for your withdrawal.

This study aims to propose a model of orderly integration of traditional therapists into the national health system, which would be a benefit for the many patients who use TM but also for practitioners of both conventional and traditional medicines, who will be able to enrich each other in complementarity. For their part, health authorities will be able to better regulate the TM practice that escapes them currently.

Principal Investigator name: ……………………

Date and signature: ....

Respondent name (optional): ....

Date and signature: ....
